# Supplementary material for: Crystal Structure of the Human, FIC-Domain Containing Protein HYPE and Implications for Its Functions
Source: Structure. 2014 Dec 2;22(12):1831–43. doi: 10.1016/j.str.2014.10.007 (PMC4342408; doi:10.1016/j.str.2014.10.007)
Supplement: Document S1. Supplemental Experimental Procedures and Figures S1–S7 [file mmc1.pdf]

Structure, Volume *22*

Supplemental Information

Crystal Structure of the Human, FIC-Domain Containing Protein HYPE and Implications for Its Functions

Tom D. Bunney, Ambrose R. Cole, Malgorzata Broncel, Diego Esposito, Edward W. Tate, and Matilda Katan

```
[Caenorhabditis    MSVRRRT---HSDDFSYL-------------LEKTRRPSK-----LN----VVQEDPKSA
[Drosophila        ----MGT---EAEQPSPPAQQQDQEN---PPLCKAQNPKP--------------------
[Xenopus           ---------------------------------------------------MAVTECEWA
[Gallus            MAERGGRGREEAGQPGWSRQQEAEGGGQGRGSTKGDVPRIRAGSRMNLISMATEPELKWV
[Mus               ------------------------------------------MILMPMASVVAVAEPKWV
[Homo              ------------------------------------------MMLIPMASVMAVTEPKWV

[Caenorhabditis    PPQGYSLTTVIIISVLVSLICQHFVPYAVST--LHTVIKNSPKQKSSPPPSNRLNIGFIS
[Drosophila        ----ARLYRFVLIFVAGSLAAWTF-HALSSTN-LVWKLRQLHHLPTAHYLQTRDEFALYS
[Xenopus           SLGSRIGLRAALVLLSGSLLVVLF-PLSGLEHQYRTALNIL------LQCN---LWGGDD
[Gallus            ALWVRVRWAAVLVLLLGSLLLLLL-PLPAVEEKCHAMLRGL------SFLRSKLGTGYTG
[Mus               SVWGRFLWMALLSMALGSLLALLL-PLGVVEEHCLAVLRGF------HLLRSKLDRAQLV
[Homo              SVWSRFLWVTLLSMVLGSLLALLL-PLGAVEEQCLAVLKGL------YLLRSKPDRAQHA
                              :     **    :             :.                 .

[Caenorhabditis    GNSPEKYAPAVQKPTFLVDPIYDEKWKGIQTAVPVMSTQ---TDEKRENDPAKVKEAILA
[Drosophila        VEE-------------------LNAFKEFYDKSVSDSVGAS----YTEAEQTNIKEALGA
[Xenopus           R----------------------HTFTGQTRGLAVASTAIELLVLKQKPTSDVKFEAKAA
[Gallus            V----------------------TRYTGQSTGLSVTSQGLELLVLKGKASPEVKLEAKAA
[Mus               V----------------------PKCTSLCTELSVSSRDAGLLTVKTTASPAGKLEAKAA
[Homo              A----------------------TKCTSPSTELSITSRGATLLVAKTKASPAGKLEARAA
                                             .         *                  **  *

                    TPR 1 α1        TPR 1 α2         TPR 2 α1          TPR 2 α2
[Caenorhabditis    AKAAGRSRKDGNLERAMTIMEHAMALAPTNPQILIEMGQIREMH-NELVEADQCYVKALA
[Drosophila        LRMAQDLYLAGKDDKAARLFEHALALAPRHPEVLLRYGEFLEHNQRNIVLADQYYFQALT
[Xenopus           LNQALEMKRQGKKEKAHKLLHHALKMDPDHVDALNELGILLEEE-KDIIQADYLYSKALT
[Gallus            LNQALEMKRQGKREKAHKLFVYALKMDPDYVDALNEFGIFSEEE-KDILQADYLYSKALT
[Mus               LNQALEMKRQGKRGKAHKLFLHALKMDPGFVDALNEFGIFSEED-KDIIQADYLYTRALT
[Homo              LNQALEMKRQGKREKAQKLFMHALKMDPDFVDALTEFGIFSEED-KDIIQADYLYTRALT
                    . *      *:  :*  :: :*: : *   : * . * : * . .::: **  * :**:

                                   Linker α                            FIC α inh
[Caenorhabditis    YDPGNSEALVLRARTTPLVSAIDRKMLRSVHDLRDEFNHL-QHSTALRRMMRETYFLYVY
[Drosophila        ISPSNSEALANRQRTADVVQSLDERRLESLDSKRDALSAIHESNGALRRAKKEAYFQHIY
[Xenopus           ISPHNEKALINRDRTLPLVEEIDQRYFSLIDSKVKKLMSIPKGNPALRRVMEESYYHHIY
[Gallus            LSPCNEKALINRDRTLPLVEEIDQRYFSIIDSKVKKVMAIPKGNSALRRVMEESYYHHIY
[Mus               ISPFHEKALVNRDRTLPLVEEIDQRYFSVIDSKVKKVMSIPKGSSALRRVMEETYYHHIY
[Homo              ISPYHEKALVNRDRTLPLVEEIDQRYFSIIDSKVKKVMSIPKGNSALRRVMEETYYHHIY
                    .* ..:**  * **  :*. :*.: :  :..  . .  : : . ****  .*:*: ::*

                               FIC α pre A             FIC α pre B
[Caenorhabditis    HTVAIEGNTLSLGQTRAILESGMVIPGKSIREHNEVIGMDAALRFLNCSLLSKEHDEISI
[Drosophila        HSVGIEGNTMTLAQTRSILETRMAVDGKSIDEHNEILGMDLAMKYINASLVQK-ID-ITI
[Xenopus           HTVAIEGNTLSLSEIRHIIETRYAVPGKSLEEQNEVIGMHAAMKYVNATLVSR-IGSVTI
[Gallus            HTVAIEGNTLTLSEIRHIIETRYAVPGKSLVEQNEVIGMHAALKYVNTTLVSR-IGSVTI
[Mus               HTVAIEGNTLTLSEIRHILETRYAVPGKSLEEQNEVIGMHAAMKYINTTLVSR-IGSVTM
[Homo              HTVAIEGNTLTLSEIRHILETRYAVPGKSLEEQNEVIGMHAAMKYINTTLVSR-IGSVTI
                   *:*.*****::*.: * *:*:  .: ***: *:**::**. *::::* :*:.:    :::

                       FIC α1                  FIC - flap     FIC α2
[Caenorhabditis    DDILEMHRRVLGNADPVEAGRIRTTQVYVGRFTPVSPEYVMEQLKDIVDWLNDESTLTID
[Drosophila        KDILELHRRVLGHVDPIEGGEFRRNQVYVGGHIPPGPGDLALLMQRFERWLNSEHSSTLH
[Xenopus           DNILEIHRRILGYVDPVEAGRFRRNQVFVGHHIPPHPRDVEKLMQEFVQWLNSEEAMSLH
[Gallus            TDILEIHRRVLGYADPVEAGRFRTTQVFVGHHIPPHPQDVGKQMQEFVQWLNSEDAMSLH
[Mus               DDMLEIHRRVLGYVDPVEAGRFRRTQVLVGHHIPPHPRDVEKQMQEFTQWLNSEDAMNLH
[Homo              SDVLEIHRRVLGYVDPVEAGRFRTTQVLVGHHIPPHPQDVEKQMQEFVQWLNSEEAMNLH
                    ::**:***:** .**:*.*.:* .** ** . *  *  :   :: :  ***.* : .:.

                      FIC α3                FIC α4                FIC α post A
[Caenorhabditis    PIERAAIAHYKLVLVHPFTDGNGRTARLLLNLIMMRSGFPPVILPVETRAEYYASLHVAN
[Drosophila        PVNYAALAHYKLVHIHPFVDGNGRTSRLLMNTLLMRAGYPPVIIPKQQRSKYYHFLKLAN
[Xenopus           PVEFAALAHYKLVYIHPFVDGNGRTSRLLMNLILMQAGYPPITVRKEQRSEYYHVLEIAN
[Gallus            PVEFAALAHYKLVYVHPFIDGNGRTSRLLMNLILMQAGYPPITIRKEQRAEYYHVLEVAN
[Mus               PVEFAALAHYKLVYIHPFIDGNGRTSRLLMNLILMQAGYPPITIRKEQRSEYYHVLEVAN
[Homo              PVEFAALAHYKLVYIHPFIDGNGRTSRLLMNLILMQAGYPPITIRKEQRSDYYHVLEAAN
                   *:: **:****** :*** ******:***:* ::*::*:**: :  : *:.**  *. **

                                    FIC α post B
[Caenorhabditis    LGDLRPFVRYVAKHSEASIQRYIGAMKTSSDNILNSGDSKLTPEESEVSEKIEAECRA--
[Drosophila        EGDIRPFVRFIADCTEKTLDLYLWATSDLPQQIPM-----LIQTESEAGERLAQMQSPNV
[Xenopus           EGDVRPFIRFIAKCTESTLDLLLIATAEHPVGLPE-----PNHGFSECKQTITIKT----
[Gallus            EGDVRPFIRFIAKCTETTLDMLLIATTEYSVGLPE-----ADGSSAGCKQTIPVKT----
[Mus               EGDVRPFIRFIAKCTEVTLDTLLLATTEYSVALPE-----AQPNHSGFKETLPVRP----
[Homo              EGDVRPFIRFIAKCTETTLDTLLFATTEYSVALPE-----AQPNHSGFKETLPVKP----
                    **:***:*::*. :* :::  : *       :            :   : :

[Caenorhabditis    --------------GN--
[Drosophila        AQRSSILEFYESGSGDLP
[Xenopus           ------------------
[Gallus            ------------------
[Mus               ------------------
[Homo              ------------------
```

Figure S1, relate to Figure 1. Sequence alignment of HYPE proteins from different species. Main structural elements, α-helices and flap, described in main Figures 1 and 2 are indicated in corresponding colors. Sequences corresponding to the inhibitory motif and catalytic loop are boxed.

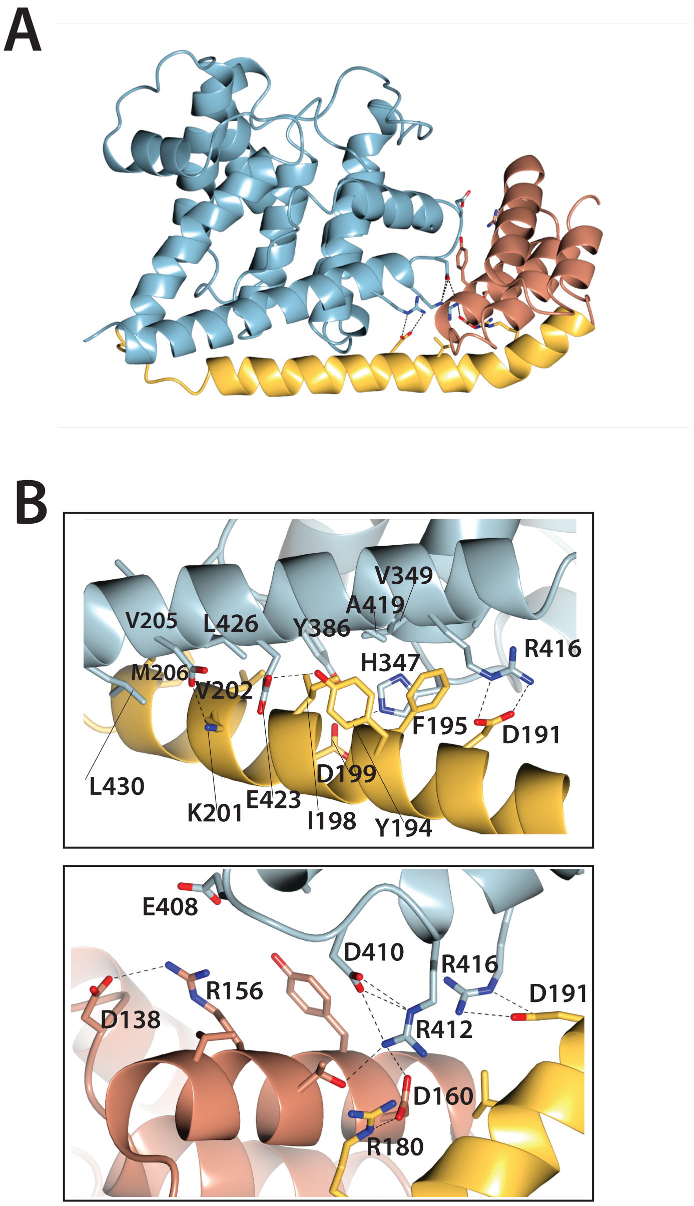

Figure S2, related to Figure 1. Surfaces of intra-molecular interactions

A. Ribbon representation of the HYPE construct; some of the residues involved in intra-molecular interactions are shown.

B. Residues involved in interactions between the FIC domain and linker are indicated in the top panel and residues contributing to interactions between the second TPR motif, FIC domain and linker are highlighted in the bottom panel.

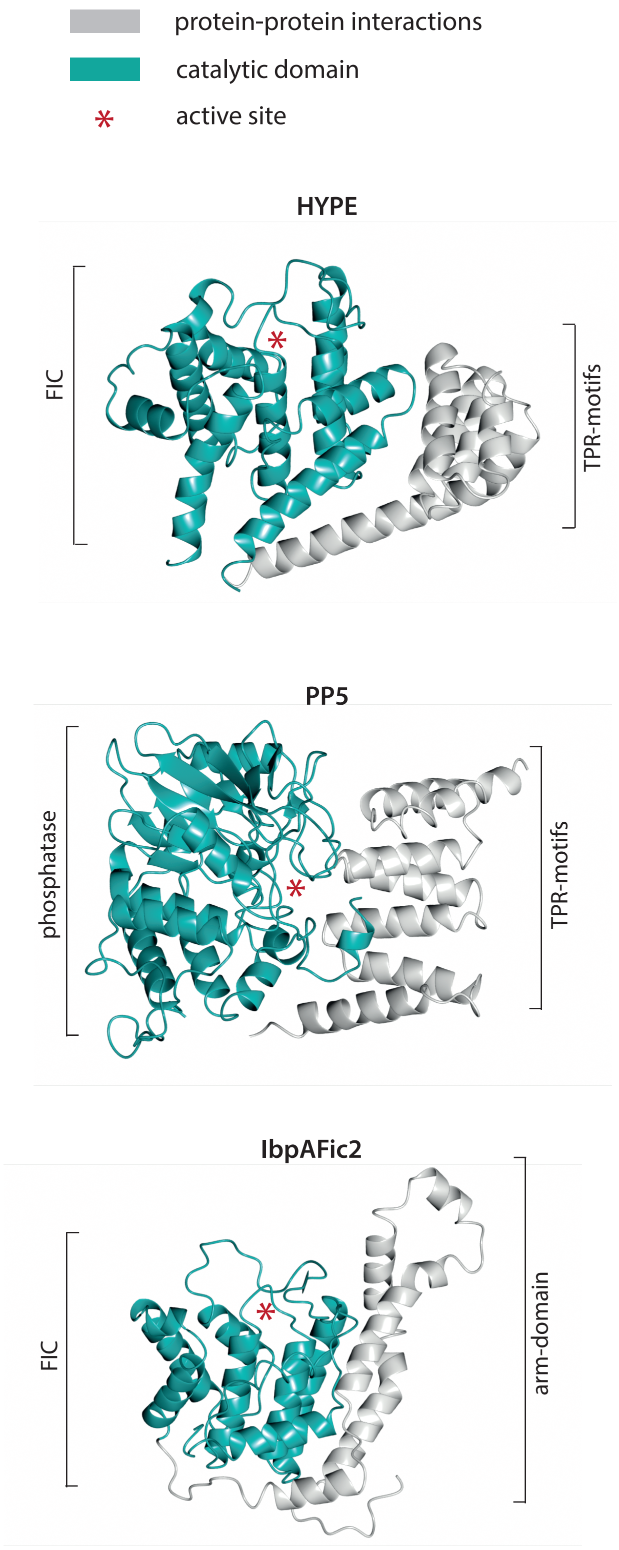

Figure S3, related to Figure 1. Inter-domain interactions in HYPE, PP5 and IbpAFic2
Relative orientation of the FIC domain and TPR-motifs in HYPE (top) is compared to orientation of phosphatase domain and TPR-motifs in PP5 (middle) and the FIC and arm domains of IbpAFic2 (bottom).
* indicates active site.

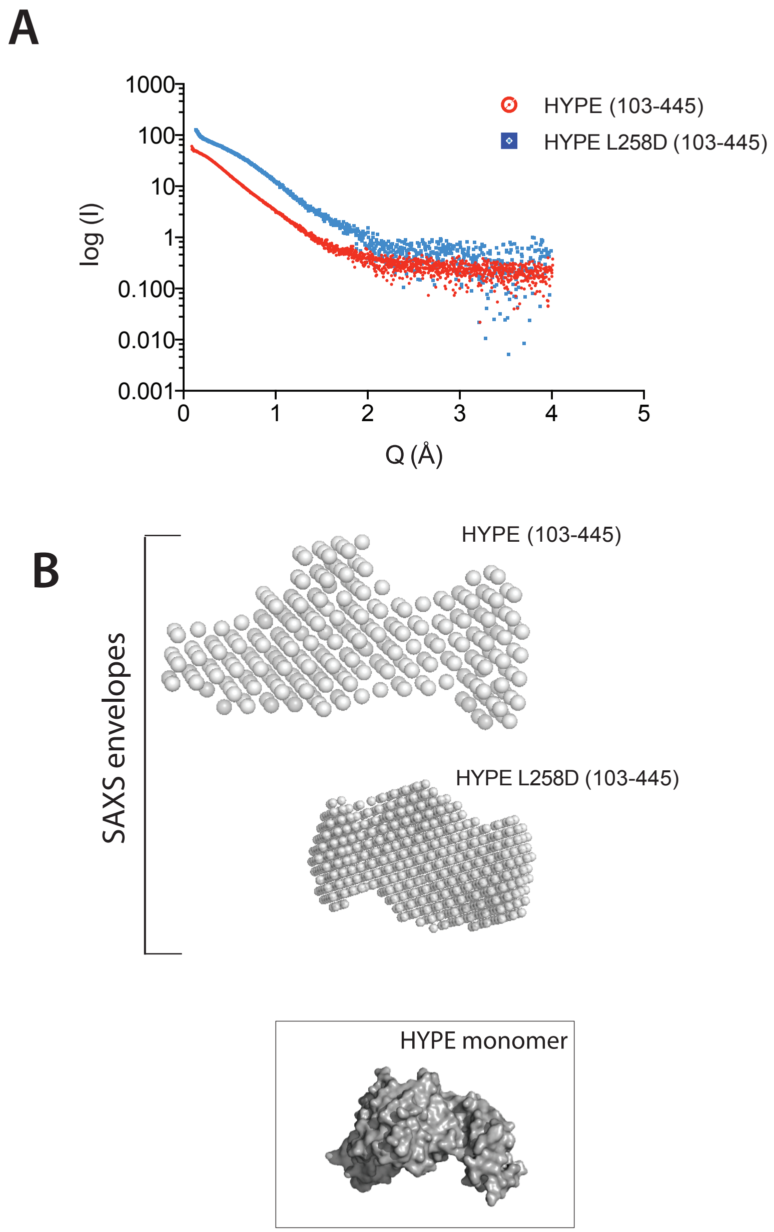

Figure S4, related to Figure 4. SAXS-based solution structure modeling of HYPE proteins
A) Log of scattered intensity versus Q for the experimental SAXS data of wild type HYPE (red circles) and the L258D variant (cyan squares).
B) DAMMIN generated envelopes of the wild type HYPE construct and L258D variant (top). Surface representation of the crystal structure of HYPE monomer is shown using the same relative scale as for the envelopes (bottom).

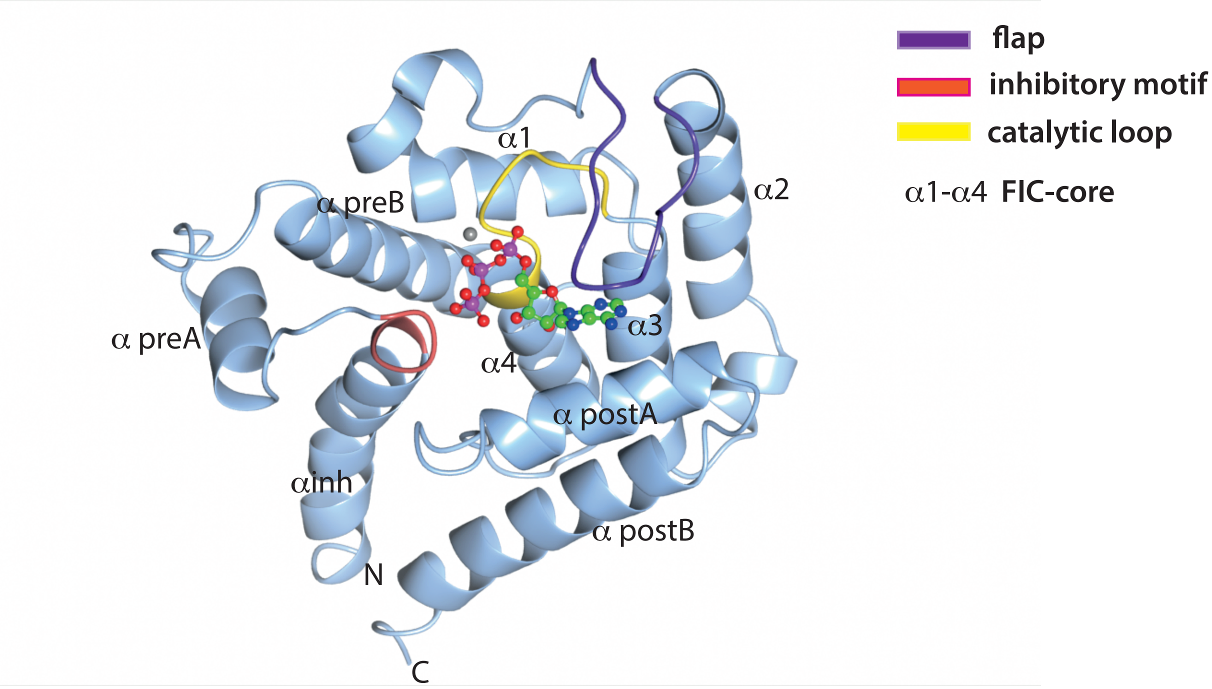

Figure S5, related to Figure 2. Features of FIC domain from HYPE
Structure of the FIC domain with highlighted catalytic loop, flap and inhibitory motif. ATP binding to E234G variant is shown; with respect to other structural features, no other changes outside the ATP binding site were observed in the corresponding structure, lacking this mutation.

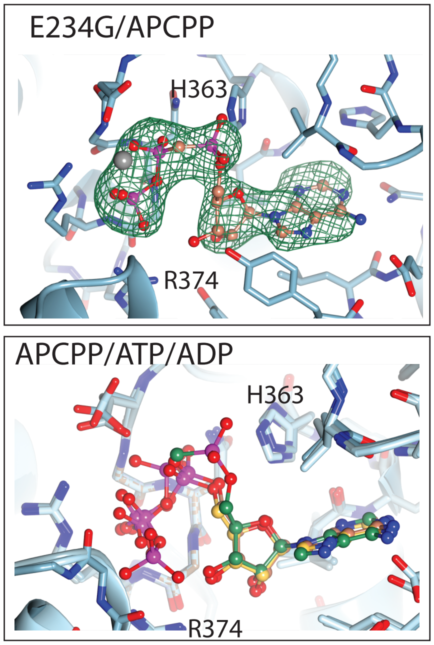

Figure S6, related to Figure 5. Binding site of APCPP
A) Binding pocket for APCPP in the E245G HYPE variant; density of APCPP, catalytic His 363 and γ-phosphate coordinating Arg 374 are indicated.
B) Overlay of ATP, ADP and APCPP in the binding pocket of E234G variant

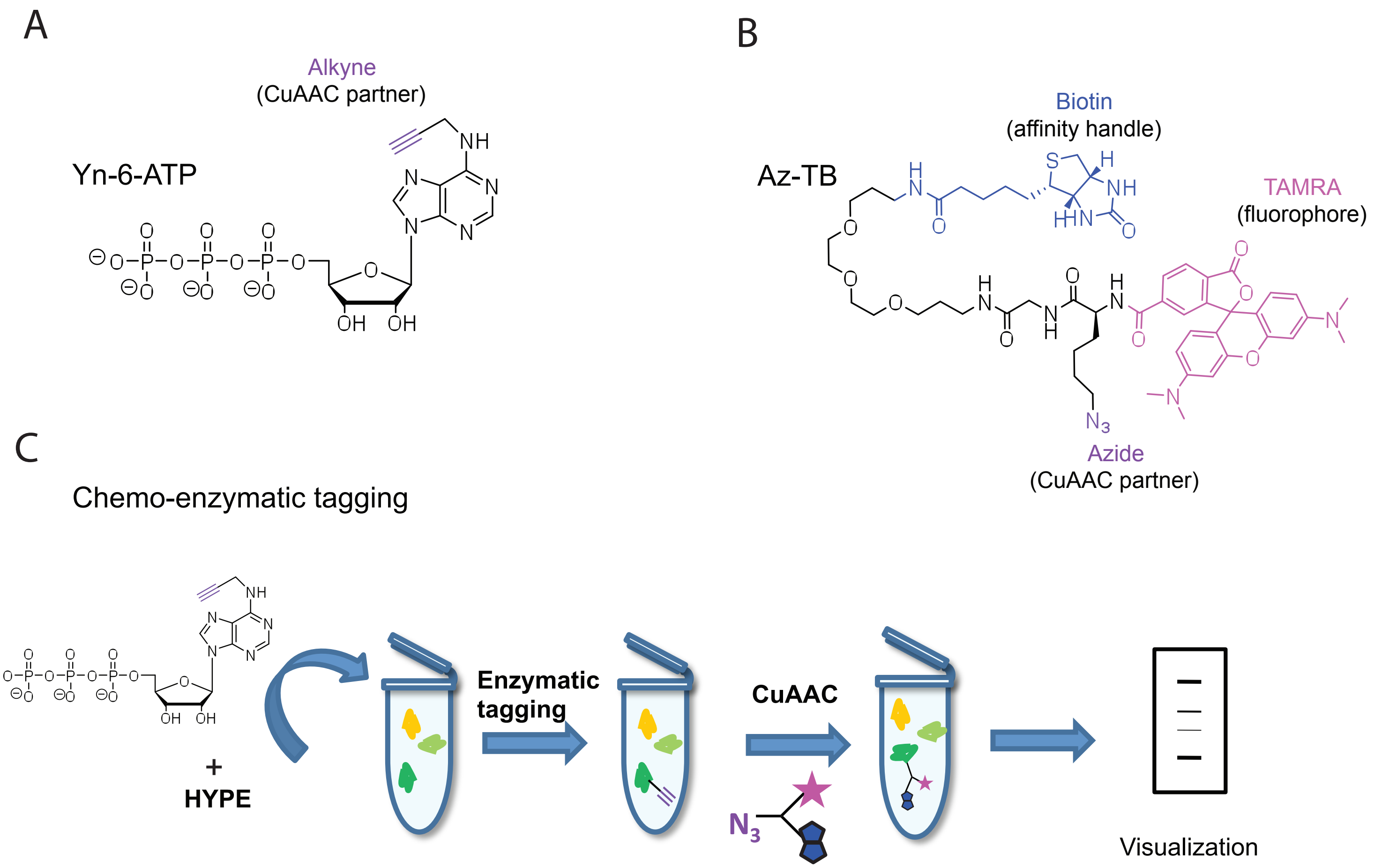

Figure S7, related to Figure 7. AMPylation assay
A) Structure of alkyne tagged adenosine triphosphate analogue Yn-6-ATP
B) Structure of multi-label reagent Az-TB used in CuAAC reaction; the fluorophore (TAMRA) permits direct in-gel detection, whilst biotin enables enrichment of AMPylated targets on streptavidin-agarose beads.
C) Auto-AMPylation or AMPylation of target proteins results in their tagging; the tagged proteins are ligated by copper-catalysed azide-alkyne cycloaddition (CuAAC) to a multi-label reporter that allows enrichment and visualization.

Supplemental Experimental Procedures

Western blotting of full-length HYPE constructs expressed in HEK 293F cells

Transfected Freestyle 293 cells were pelleted in 1 ml aliquots and the protein extracted in 0.25 ml of 25 mM Tris.Cl, 150 mM NaCl, 1 mM TCEP, 1 %(v/v) Triton-X-100, pH 7.5 containing a Complete protease inhibitor tablet (Roche). Protein concentration were determined following the BCA method and 30 µg loaded on an SDS-PAGE minigel. Proteins were transferred to PVDF membrane and blotted with an antibody against AMP-Threonine (Millipore). Blots were developed using ECL and film.

Small angle X-ray scattering (SAXS)

Synchrotron SAXS data were collected at ESRF (Grenoble). All samples were measured in 25 mM Tris.Cl, 150 mM NaCl, 5 mM TCEP, 10 %(v/v) glycerol at pH 8.0. Proteins were measured at 1, 2 and 5 mg/ml concentrations. The data were processed by the program PRIMUS (Konarev et al., 2003) following standard procedures to compute the radii of gyration (Rg) and maximum dimensions (Dmax) (Svergun and Koch, 2002). The distance distribution functions, p(r), were evaluated using the program GNOM (Svergun, 1992). The SAXS data for the monomeric mutant showed evidence of the formation of large aggregates apparent at lower q-values. We therefore excluded the points before the linear guiner region and used the rest of the curve for further modelling.

Low-resolution models were generated by the *ab initio* program DAMMIN (Svergun, 1999), which represents a protein by volume filled with the packed spheres referred to as dummy atoms. The results of 10 independent DAMMIN runs were analyzed and averaged by DAMAVER (Volkov and Svergun, 2003) and overlapped to the available crystal structure with the program SUPCOMB (Kozin and Svergun, 2001).

AMPylation assays

Cell culture and lysis. HeLa cells were cultured in a humidified atmosphere of 10% CO_2 at 37° C in DMEM supplemented with 10% FBS. Prior to lysis cells were washed twice with PBS and

harvested into the lysis buffer (20 mM Hepes pH 7.4, 100mM NaCl, 5 mM $MgCl_2$, 1% Triton X-100) supplemented with EDTA-free protein inhibitor (Roche). Next, the cells were disrupted by sonication and cellular debris was pelleted by centrifugation (17,000 g, 10 min). Protein concentration was measured with BCA assay (BioRad).

AMPylation reaction. Auto-AMPylation of recombinant HYPE variants (2 µg each) was carried out in the lysis buffer supplemented with BSA (2 µg), DTT (1 mM) and Yn-6-ATP (100 µM; Jena Bioscience) for 1 h at 30° C. AMPylation in cell lysates (50 µg protein) was performed using recombinant AMPylators (5 µg) as described above. AMPylation was stopped by protein precipitation (chloroform/methanol, 0.25:1, relative to the sample volume). Precipitates were isolated by centrifugation (17, 000 g, 10 min), washed once with methanol (400 µL) and air dried (10 min). Dried pellets were then resuspended (PBS, 0.2% SDS) at 1 mg/mL for the 'click' reaction.

CuAAC and in-gel fluorescence. A click mixture was prepared by adding reagents in the following order and by vortexing between the addition of each reagent: Az-TB (0.1 mM), $CuSO_4$ (1 mM), TCEP (1 mM), TBTA (0.1 mM). Az-TB was synthesized as described in Heal et al. (Heal et al., 2012). Following the addition of the click mix, samples were vortexed at RT for 1 h. The reaction was stopped by addition of EDTA (final concentration 10 mM) and subsequently proteins were precipitated, washed and dried as described above. The dried pellets were resuspended (PBS, 2 % SDS) and 4× LDS sample loading buffer (Invitrogen) was added (final concentration of proteins 1 mg/mL). The samples were then boiled (5 min), centrifuged (1,000 g, 2 min) and loaded on SDS-PAGE gel. After the run, the gel was washed with MilliQ (3x), soaked in fixing solution (40 % MeOH, 10 % acetic acid, 50 % water) for 5 min and washed with MilliQ (3x) again. The fluorescence on the gel was detected using an Ettan DIGE Imager (GE Healthcare) and the protein loading was checked by Coomassie.

Supplemental References

Heal, W.P., Wright, M.H., Thinon, E., and Tate, E.W. (2012). Multifunctional protein labeling via enzymatic N-terminal tagging and elaboration by click chemistry. Nat Protoc *7*, 105-117.

Konarev, P.V., Volkov, V.V., Sokolova, A.V., Koch, M.H.J., and Svergun, D.I. (2003). PRIMUS: a Windows PC-based system for small-angle scattering data analysis. J Appl Crystallogr *36*, 1277-1282.

Kozin, M.B., and Svergun, D.I. (2001). Automated matching of high- and lowresolution structural models. J Appl Crystallogr *34*, 33-41.

Svergun, D.I. (1992). Determination of the Regularization Parameter in Indirect-Transform Methods Using Perceptual Criteria. J Appl Crystallogr *25*, 495-503.

Svergun, D.I. (1999). Restoring low resolution structure of biological macromolecules from solution scattering using simulated annealing. Biophys J *76*, 2879-2886.

Svergun, D.I., and Koch, M.H. (2002). Advances in structure analysis using small-angle scattering in solution. Curr Opin Struct Biol *12*, 654-660.

Volkov, V.V., and Svergun, D.I. (2003). Uniqueness of ab initio shape determination in small-angle scattering. J Appl Crystallogr *36*, 860-864.
