# Supplementary material for: Crystal Structure of the Human, FIC-Domain Containing Protein HYPE and Implications for Its Functions
Source: Structure. 2014 Dec 2;22(12):1831–43. doi: 10.1016/j.str.2014.10.007 (PMC4342408; doi:10.1016/j.str.2014.10.007)
Supplement: Document S2. Article plus Supplemental Information [file mmc2.pdf]

Structure
Article

Crystal Structure of the Human, FIC-Domain Containing Protein HYPE and Implications for Its Functions

Tom D. Bunney,[1,5,*] Ambrose R. Cole,[2,5] Malgorzata Broncel,[3] Diego Esposito,[4] Edward W. Tate,[3] and Matilda Katan[1,*]
[1]Division of Biosciences, Institute of Structural and Molecular Biology, University College London, Gower Street, London WC1E 6BT, UK
[2]Institute of Structural and Molecular Biology, Birkbeck College, London WC1 7HX, UK
[3]Department of Chemistry, Imperial College London, South Kensington Campus, Exhibition Road, London SW7 2AZ, UK
[4]Division of Molecular Structure, MRC-National Institute for Medical Research, Mill Hill, London NW7 1AA, UK
[5]Co-first author
*Correspondence: t.bunney@ucl.ac.uk (T.D.B.), m.katan@ucl.ac.uk (M.K.)
http://dx.doi.org/10.1016/j.str.2014.10.007
This is an open access article under the CC BY license (http://creativecommons.org/licenses/by/3.0/).

SUMMARY

Protein AMPylation, the transfer of AMP from ATP to protein targets, has been recognized as a new mechanism of host-cell disruption by some bacterial effectors that typically contain a FIC-domain. Eukaryotic genomes also encode one FIC-domain protein, HYPE, which has remained poorly characterized. Here we describe the structure of human HYPE, solved by X-ray crystallography, representing the first structure of a eukaryotic FIC-domain protein. We demonstrate that HYPE forms stable dimers with structurally and functionally integrated FIC-domains and with TPR-motifs exposed for protein-protein interactions. As HYPE also uniquely possesses a transmembrane helix, dimerization is likely to affect its positioning and function in the membrane vicinity. The low rate of autoAMPylation of the wild-type HYPE could be due to autoinhibition, consistent with the mechanism proposed for a number of putative FIC AMPylators. Our findings also provide a basis to further consider possible alternative cofactors of HYPE and distinct modes of target-recognition.

INTRODUCTION

It is well established that posttranslational modifications (PTM) of proteins provide a key mechanism for control of protein functional states, protein-protein interactions, subcellular localization, and stability (Deribe et al., 2010; Kamath et al., 2011). In addition to the best-understood PTM, phosphorylation of proteins, several other common modifications have been identified including methylation, acetylation, and ubiquitination. Very recently AMPylation of eukaryotic proteins was also documented (Yarbrough and Orth, 2009). AMPylation (or adenylylation) is the transfer of AMP from ATP to a Tyr or Thr/Ser residue in target proteins. Most enzymes known to catalyze AMPylation are bacterial effectors that are secreted into infected cells, where they AMPylate small GTPases (Rho and Rab families), causing disruption to the host cell (Müller et al., 2010; Roy and Mukherjee, 2009; Yarbrough et al., 2009). These bacterial effectors are regarded as potential new targets in drug discovery since AMPylation plays an important role in infection (Lewallen et al., 2014).

The majority of bacterial AMPylators incorporate a so-called filamentation induced by cyclic AMP (FIC) domain responsible for AMP transfer (Broncel et al., 2012; Garcia-Pino et al., 2014). Further analysis of bacterial effectors has shown that the cofactor specificity is not restricted to ATP, with some FIC domains catalyzing GMPylation and UMPylation reactions (Feng et al., 2012). Furthermore, FIC domains can also catalyze other reactions instead of NMPylation, such as phosphorylation and phosphocholine transfer (Campanacci et al., 2013; Castro-Roa et al., 2013; Cruz et al., 2014). Nevertheless, as clearly illustrated for phosphocholine transfer by AnkX (Campanacci et al., 2013), the underlying reactions share a common mechanism and involve the transfer of a part of a pyrophosphate-bond-containing metabolite and the cleavage of this bond.

The first reports of AMPylation focused on the structure and function of bacterial FIC proteins (Campanacci et al., 2013; Engel et al., 2012; Feng et al., 2012; Goepfert et al., 2013; Ham and Orth, 2011; Müller et al., 2010; Roy and Mukherjee, 2009; Worby et al., 2009; Xiao et al., 2010; Yarbrough et al., 2009). These data strongly suggest that such a modification, in particular eukaryotic AMPylation, is a reversible and regulatory PTM. However, the scope and precise physiological relevance beyond bacterial infection is currently largely unknown. Interestingly, in eukaryotic genomes only one FIC-domain containing protein has been identified to date, HYPE or FICD, and it is strongly conserved from *C. elegans* to humans (Yarbrough and Orth, 2009). Domain organization is also conserved and, in addition to the FIC domain, the protein incorporates one transmembrane helix and tetratricopeptide repeat (TPR) motifs. However, very little is known about properties of HYPE with regard to both structure and its function in any of these organisms. Some initial characterizations of HYPE suggest that its FIC domain can catalyze NMPylation, including AMPylation (Engel et al., 2012; Mattoo et al., 2011; Worby et al., 2009). The only functional insight has been recently obtained from a study on *Drosophila*, where flies lacking HYPE were viable and fertile, but blind due to compromised visual neurotransmission; the link between catalytic functionality of the FIC domain and the phenotype was also established (Rahman et al., 2012).

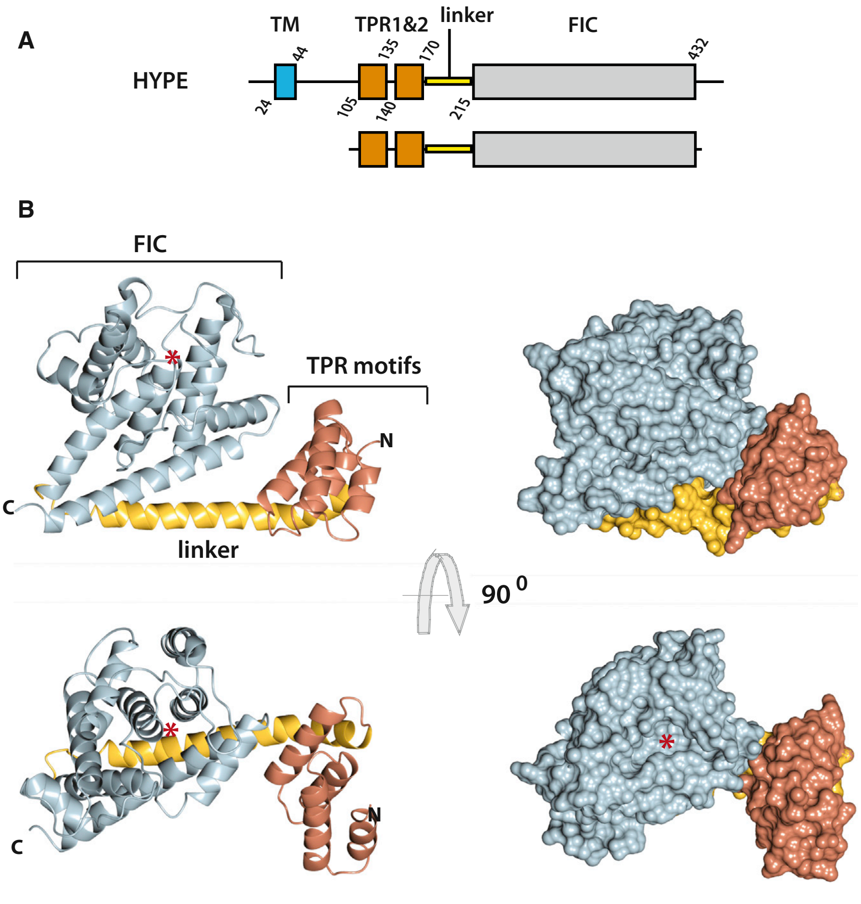

Figure 1. Crystal Structure of HYPE

(A) Schematic diagram of domain organization of full-length human protein (top) and construct used for crystallization (bottom).

(B) Ribbon (left) and surface (right) representation of HYPE structure showing relative orientations of the TPR-motifs, linker, and FIC domain. *Pocket for cofactor binding in the FIC domain of the wild-type structure.

See also Figures S1–S3.

Here we describe the first crystal structure of a eukaryotic HYPE encompassing the two TPR-motifs, an α-helical linker, and the FIC domain of the human protein. The structure and further analyses reveal several features of HYPE that are distinct from most previously characterized bacterial effectors and suggest a different cellular function for this FIC-domain protein.

RESULTS

3D Structure of the Multidomain, Human HYPE Protein

Structural studies of HYPE were performed using a construct lacking the first 102 amino-acid residues at the N terminus, incorporating a single transmembrane domain (residues 24–44). The construct included two TPR-motifs (residues 105–135 and 140–170), a linker region (residues 170– 215), and the FIC domain (residues 215–432) (Figure 1A). While the portion at the N terminus shares low sequence similarity among different species, sequences within the boundaries of the construct used for structural studies are strongly conserved throughout its length (51% similarity) (Figure S1 available online). Structures of several variants of this multidomain HYPE construct were solved by X-ray crystallography with a resolution of up to 2.5Å (Table 1).

As shown in Figure 1B, almost the entire structure of the HYPE construct is composed of α helices that represent the main secondary structure element of the TPR-motifs and the FIC domain, with the linker between them consisting of a single α helix. A surface representation of the protein (Figure 1B, right) illustrates a compact structure with restricted flexibility owing to intramolecular interactions, where each of the three main structural features (TPR-motifs, linker, and FIC domain) interact with the other two.

TPR-motifs are found in a number of different organisms (from bacteria to humans), and the number of TPR repeats, each consisting of two antiparallel α helices, varies (Allan and Ratajczak, 2011; Blatch and Lässle, 1999; D'Andrea and Regan, 2003; Zeytuni and Zarivach, 2012). The presence of only two TPR-motifs in HYPE is unusual; most TPR proteins contain three or more (up to 16) TPR-motifs implicated in protein-protein interactions. The only other example of two TPR-motifs is from the propyl 4-hydroxylase (P4H) α subunit (Pekkala et al., 2004). The structure of the TPR-motifs present in HYPE superimposes well with the structures from P4H, as well as with the more typical three-TPR domain of protein phosphatase 5 (PP5) (Figure 2A). As in other examples, parallel packing of adjacent TPR-motifs generates a right-handed helical conformation, creating a channel (or groove) that can accommodate a polypeptide from another protein (Figure 2A). In many cases, the TPR domains present an additional "capping/solubility" helix C-terminal to the TPRs. In HYPE, it is likely that the linker α helix could have this role and could be considered as a part of the TPR domain (Figure 2A). The initial TPR repeat in all HYPE structures exhibits a high degree of disorder when compared to the rest of the structure; this is reflected in the high thermal-factors for this region and missing side chains where the density is poor. Electron density is improved for the underlying main chain positions, giving confidence in the overall orientation of the helices (Table 1).

The FIC domain of HYPE shares general features of other FIC domain structures as shown by an overlay with the FIC domain from *H. pylori* and IbpAFic2 (Figure 2B). As defined by Pfam, the common core of the FIC domain includes four α helices (α1–4) (Finn et al., 2010). Additional helices at the N- and C-termini of the core are present in most FIC protein structures and show considerable variation in location and orientation. Several such additional α helices are present in HYPE, three at the N terminus and two at the C terminus. Within the FIC core, two features are present in most known structures: (1) the catalytic loop and (2) the flap (Garcia-Pino et al., 2014) (Figure 2B).

Table 1. Data Collection and Refinement Statistics

	HYPE Construct/Cofactor				
	Apo	E234G-APCPP	E234G-ATP	E234G-ADP	WT-ADP
Synchrotron	Diamond I24	Diamond I03	Soleil	home source	ESRF-ID23-1
Strategy	line scan	line scan	standard	standard	standard
Resolution (Å)	2.48A (2.61–2.48)	2.98 (3.06–2.98)	2.7 (2.84–2.7)	2.54 (2.65–2.54)	2.98 (3.25–2.98)
Rmrg	0.088 (0.854)	0.159 (0.496)	0.084 (0.57)	0.12 (0.814)	0.197 (0.41)
Mn (I/sd)	6.1 (1.0)	4.4 (1.7)	7.1 (1.7)	6.5 (0.9)	11.8 (5.3)
Comp (%)	97.7 (97.7)	94.7 (94.7)	98.1 (92.2)	98.7 (98.7)	84.0 (43.5)
Mult	3.1 (3.0)	3.5 (3.1)	3.0 (3.0)	3.2 (2.5)	14.0 (7.2)
CC_Imean	0.994 (0.701)	0.981 (0.843)	0.991 (0.815)	0.994 (0.546)	0.993 (0.718)
Spg	P21	P1	P21	P21	P21212
Mosaicity	0.18	0.34	0.17	0.94	0.2
Cell (°)	71.19, 76.81, 93.19	77.1, 83.75, 130.02	71.25, 76.11, 92.2	71.04, 76.01, 92.03	77.86, 109.08, 131.54
(Å)	90, 108.05, 90	89.92, 89.56, 89.43	90.0, 107.28, 90.0	90.0, 107.56, 90.01	90.0, 90.0, 90.0
Wilson B ($Å^2$)	63.521	39.31	43.068	68.8	51.9
PHASER model	3CUC				
Refinement					
No. reflections	33,248	65,073	27,626	32,508	20,620
R_{fac}/R_{free}	21.24/24.42	0.21/0.25	0.20/0.26	0.21/24	0.2/0.26
No. atoms					
Protein	5,053	20,412	5,051	5,062	5,172
Ligand	216	1,080	324	225	244
Water	158	824	198	145	156
B factors ($Å^2$)					
Protein	81.3	61.83	70.8	64.55	32.5
Ligand	101.25	72.6	69.65	21.92	38.55
Water	62.985	37.3	59.11	48.87	21.56
Root-mean-square deviations					
Bond lengths (Å)	0.01	0.01	0.01	0.01	0.01
Bond angles (°)	1.16	1.08	1.05	1.2	1.16

The catalytic loop in HYPE proteins, commonly positioned between the core α helices 3 and 4, shares the general signature motif of FIC domains, HxFx(D/E)(A/G)N(G/K)R, represented in HYPE by the sequence HPF(I/V)DGNGRT(S/A)R. The critical His residue within the catalytic motif corresponds to His 363 in the human HYPE (Figure S1). The second feature, the flap, is either a β-hairpin or a loop preceding helix α2; this structure appears to facilitate positioning of the target residue. In HYPE, a loop region between residues 311–324 corresponds to the flap-like structure. A recent exhaustive bioinformatics analysis coupled with homology modeling of FIC domains revealed another feature, an inhibitory motif outside the FIC core (Engel et al., 2012). The inhibitory helix (αinh) contains a common inhibitory signature, (S/T)xxxE(G/N), conserved in HYPE proteins as (T/S)V(A/G)IEN, with the critical Glu residue corresponding to Glu 234 in the human protein (Figures 2B and S1). However, some FIC domain proteins lack αinh, as illustrated here for IbpA2FIc (Figure 2B); the highly similar Vop S protein also lacks αinh (Engel et al., 2012).

The FIC domain has been predicted to be present in many (about 3,000) proteins encoded by all genomes sequenced to date, varying in length and domain organization (Kinch et al., 2009). The crystal structure of HYPE (Figures 1 and S2A) reveals the interaction surfaces between the FIC domain and the linker α helix, and between the FIC domain and α helix 2 of the second TPR-motif (TPR2 α2). The FIC domain interaction surface with the linker is more extensive (≈684 $Å^2$) and contributes residues mainly from the post B α helix; however, residues from α3 and αpost A linker (flanking the catalytic loop), as well as one residue from αinh, are also involved. The contact area between the FIC domain and TPR2 α2 is smaller (≈215 $Å^2$) and restricted to the αpost A and αpost B helices of the FIC domain. The N-terminal portion of the linker also interacts with the TPR2 motif (with residues within TPR2 α1 and α2 helices). Overall, these intramolecular interactions likely result in restricted flexibility for the molecule, where the catalytic site of the FIC domain and TPR-motifs are accessible to cofactor binding and protein-protein interactions, respectively (Figures 1 and S3).

Dimerization of HYPE

The crystal structure of HYPE revealed asymmetric dimers with an interaction surface formed exclusively of FIC domain contacts (Figure 3A). There are two distinct areas of interactions (Figure 3B). The first encompasses αpre A helix, αpre B helix, and

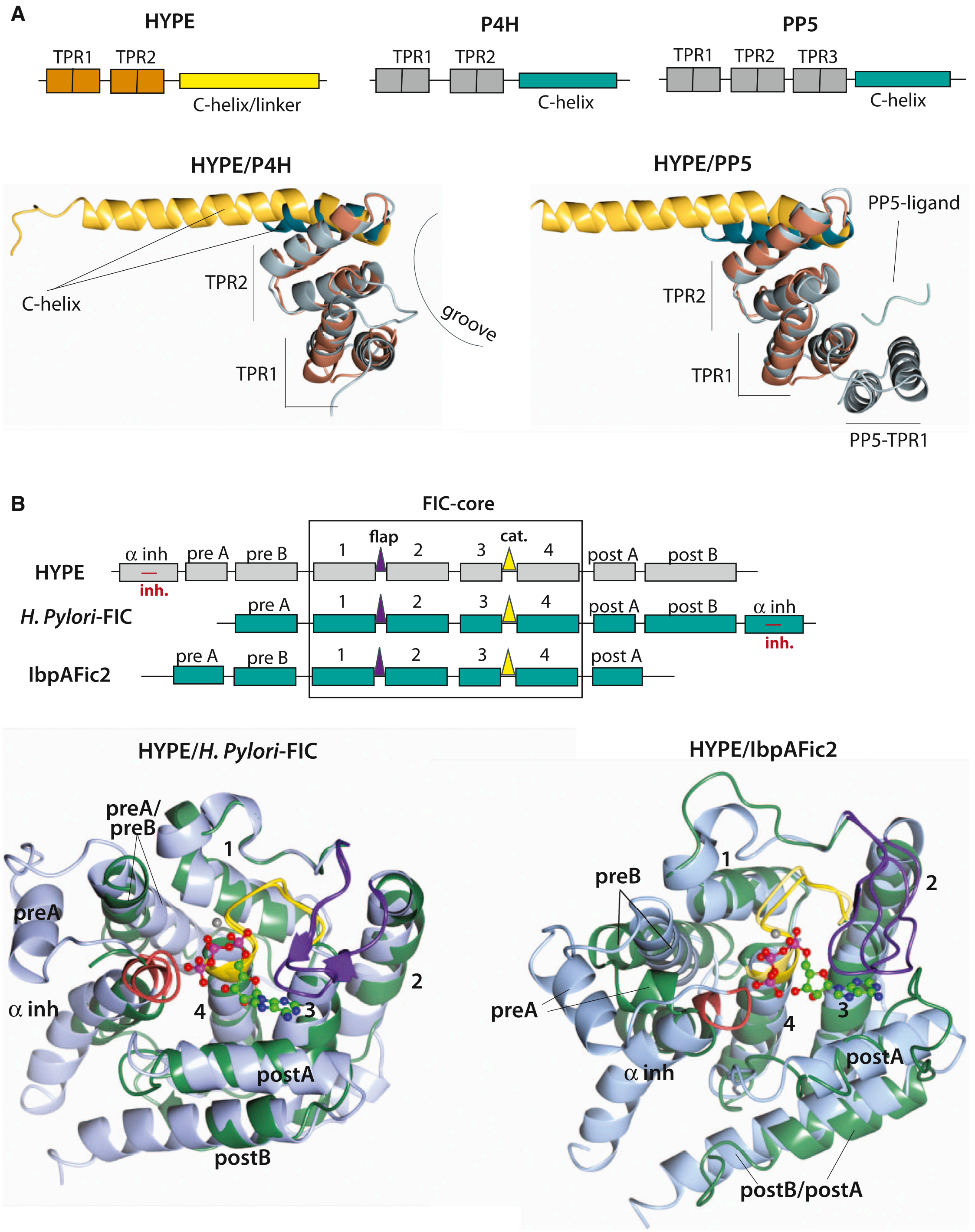

Figure 2. Features of TPR and FIC Domains

(A) Schematic diagrams (top) and structural comparison (bottom) of TPR domain from HYPE with P4H TPR domain (left, 1TJC) and PP5 TPR domain (right, 2BUG). Position of a peptide from Hsp 90, binding to the TPR groove in PP5 is also shown (right).

(B) Schematic diagrams (top) and structural comparison (bottom) of FIC domain from HYPE with *H. Pylori* FIC (left, 2F6S) and IbpA2Fic (right, 4ITR). Positions of the catalytic loop (yellow), flap (purple), and inhibitory motif (red) are shown, as well as the position of ATP-cofactor from structure of E234G HYPE. FIC-domain core (FIC-core) α helices are labeled as 1–4. The last, αinh helix from *H. Pylori* FIC is circularly permuted and overlays with the αinh helix from HYPE FIC. IbpAFic2 lacks an αinh helix. Pre B α helix from HYPE FIC overlays with pre A α helix from *H. Pylori* FIC, while post B α helix from HYPE FIC overlays with post A α helix from IbpAFic2. See also Figure S5.

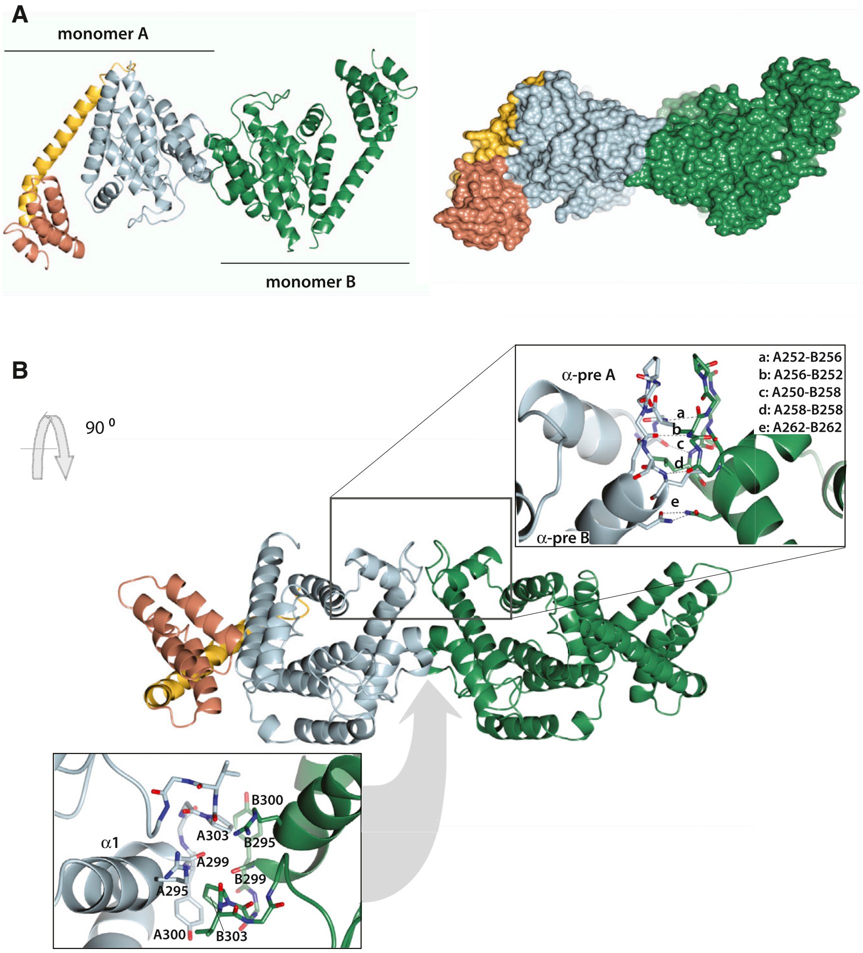

Figure 3. HYPE Dimers and the Dimer Interface

(A) Ribbon (left) and surface (right) representation of crystal structure of HYPE dimers. Structure of the wild-type HYPE is shown and essentially the same structure is obtained for E234G variant.

(B) The two interface areas, indicated in the ribbon representation of a HYPE dimer, are shown as insets. The amino acid residues indicated in the first area (top inset) correspond to V252, K256, Y250, L258, and N262; they are shown as residue numbers in monomers (A) and (B). The amino acid residues indicated in the second area (bottom inset) correspond to R295, G299, Y300, and D303.

their linker. The second area incudes α1 helix and follow up linker, preceding the flap. The first dimerization region is more extensive (≈450 Å^2) and involves a number a hydrogen bonds; the second area is smaller (≈375 Å^2) with weaker interactions, likely to allow for some flexibility (Figure 3B, insets).

The TPR-motifs and the linker are not involved in dimerization, and all TPR-motifs are exposed for ligand binding. Interestingly, TPR-motifs from each monomer are positioned at the opposite sides of the dimer surface (Figure 3). This arrangement precludes cooperation of TPR repeats from two monomers and would allow interactions with two protein partners, and thus contribute to formation of larger complexes. TPR recognition could also be related to selection of protein targets for PTM (see Discussion).

A further analysis of the properties of HYPE constructs in solution confirmed that dimerization is not restricted to conditions used for crystallization. Using size exclusion chromatography, we showed that the elution profile of HYPE corresponded to dimers, and as expected from the crystal structure, the deletion of TPR-motifs did not disrupt dimerization (Figure 4A). However, mutations of residues at the dimerization surface identified a single residue replacement, L258D, sufficient to generate a HYPE monomer (Figure 4A).

Further properties of the HYPE dimers and monomers were analyzed by small angle X-ray scattering (SAXS) (Figures 4B and S4). In agreement with the crystal structure, the SAXS generated wild-type model was consistent with it being a dimer in solution. In contrast, the parameters generated for the L258D HYPE variant suggest this exists as a monomer in solution.

As discussed further below, dimerization of HYPE results in two transmembrane domains per dimer of the full length HYPE and is likely to have an impact on the relative orientation toward membrane structures and further restrict flexibility (see Discussion).

FIC Domain and Cofactor Binding

There are two critical elements for the enzyme activity of FIC proteins, these are the catalytic loop and, for some FIC domains, also the inhibitory helix (Figure S5). In HYPE, the conformation of the catalytic loop is the same as in other structurally defined FIC enzymes (Garcia-Pino et al., 2014); a scaffold of two α helices (α3 and α4) together with the side chains of the conserved Phe365 and Asn369 of the catalytic loop contribute to its distinct structure. The Phe365 side chain anchors the catalytic loop to the hydrophobic core of the enzyme, whereas the amide group of the conserved Asn369 holds the loop through a network of hydrogen bonds to the peptide backbone. The GNG submotif forms an ‘‘anion hole’’. In the structure of wild-type HYPE, the inhibitory glutamate, Glu234, from αinh is positioned in the vicinity of the catalytic loop.

In addition to apo structures of HYPE variants, we have also obtained structures with the ATP cofactor bound to an E234G variant and with ADP bound to either the HYPE E234G variant or to wild-type protein (Figures 5A and 5B). The positions of the adenosine moiety, ribose ring, and phosphates from all three structures and orientations of critical residues are generally consistent with the previous insights from structural studies of complexes of other FIC domains with ATP and ATP-related ligands (Engel et al., 2012; Goepfert et al., 2013; Palanivelu et al., 2011; Xiao et al., 2010). The adenosine moiety of the ATP and ADP locks into a hydrophobic pocket formed between α3, αpost A and the flap loop; the side chain residues forming this pocket are Val360 (α3), Leu403 (αpost A), and Val316 (flap). There is also direct coordination by one residue, Asn407 (Figures

A

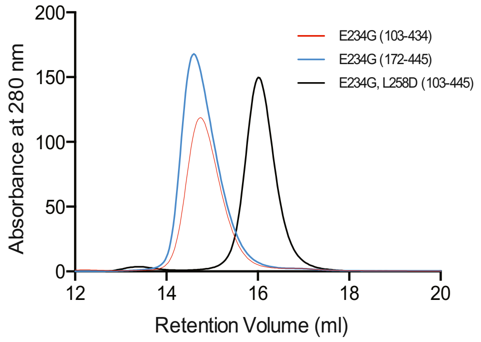

B

Sample	Theoretical Mass (kDa)	Rg (nm)	Dmax (nm)	χ^2
HYPE (103-445)	78.9	4.1 ± 0.01	12.9 ± 0.5	1.049
HYPE L258D (103-445)	39.4	2.7 ± 0.02	8.0 ± 0.5	2.484

Figure 4. Properties of HYPE Dimers in Solution

(A) Analytical gel filtration of HYPE constructs. HYPE variant (103–434), containing TPR-motifs, linker, and FIC domain, is shown in red. HYPE (172–445), containing the linker helix and FIC domain, is shown in blue. The HYPE (103–445) L258D mutant is shown in black.

(B) Selected parameters of soluble HYPE constructs from analyzed SAXS data. See also Figure S4.

5B and 5C). The GNG anion hole (residues 368–370) accommodates the α-phosphate of the nucleotide, mainly through direct hydrogen bonds to the polypeptide backbone. A Mg^{2+} ion is visible in the E234G variant of HYPE; it bridges the α- and β-phosphates and is coordinated by the conserved Asp367 side chain. The conserved arginine at the C-terminal side of the FIC catalytic loop, Arg374, forms hydrogen bonds with the ribose ring and is also critical for binding of the γ-phosphate. The position of the inhibitory glutamate (Glu234) in the structure of wild-type HYPE is consistent with its role in competing with the Arg374/γ-phospate interaction (Figure 5A). Interestingly, there is a difference in side chain orientation of Glu234 in apo and ADP bound structures of the wild-type HYPE that shows that this side chain can also affect ADP binding and that is sufficiently flexible to accommodate bound ADP (Figure 5A). The position of clearly visible α- and β-phosphates in all three structures is the same, with the α-phosphate present in an orientation compatible with the AMPylation reaction (Figure 5A). This is consistent with previous findings that the engagement of β- and γ-phosphates of ATP or AMPPNP to wild-type FIC proteins is obstructed by the inhibitory glutamate, resulting in a nonproductive orientation of the α-phosphate, while in all structures obtained for glutamate substitutions or deletions, the position of ATP is the same, with a productive orientation of the α-phosphate (Engel et al., 2012; Goepfert et al., 2013). Correct positioning of the α-phosphate in the wild-type HYPE/ADP complex shows that also in the case of HYPE, the γ-phosphate, and to some extent β-phosphate, could preclude efficient and/or enzymatically correct ATP binding to the wild-type protein.

For the transfer reaction catalyzed by FIC enzymes, the productive binding of the nucleotide is achieved by the correct positioning of the conserved Arg374; this in turn allows insertion of the attacking group of the target, facilitated by the flap structure. The conserved His363 in HYPE is well positioned to compete with the catalytic motif that would result in a favorable interaction with a high-energy pyrophosphate bond of the nucleotide to act as a general base in the catalysis, allowing the transfer of the AMP moiety (Figures 5B and 5C). The structure of HYPE E234G with the nonhydrolysable ATP analog, APCPP, shows a different position of the α-phosphate that would be incompatible with catalysis (Figure S6), suggesting that APCPP is not a physiological ATP mimic in this case.

Based on the limited coordination of adenosine in the hydrophobic pocket, it is likely that other nucleotide phosphates or other cofactors can bind to this site. Measurements of binding of a range of ligands to the E234G variant (172–434) using thermal shift analysis excluded phosphocholine as a possible cofactor (utilized by some FIC-domain proteins such as AnkX) (no shift in Tm, data not shown) and showed similar binding of ATP and guanosine-5'-triphosphate, and a less strong binding of cytidine triphosphate and uridine triphosphate (Figure 6A). Interestingly, binding of ADP was stronger than that of ATP. A possible reason could be that ATP adopts a less favorable conformation within the binding pocket compared to free ATP, a constraint that would not apply to ADP. The measurement of the dissociation constant (K_D) of ADP binding to the E234G variant determined by isothermal titration calorimetry (ITC) was 160 nM (Figure 6B). Wild-type HYPE also bound ADP (as the only ligand, Figure 6A), but with a considerably lower affinity and K_D of 1.5 μM (Figure 6B), possibly owing to electrostatic repulsion by the side chain of by Glu234. Binding of ATP to wild-type HYPE could not be detected using these methods. These findings are in general agreement with the structural data and with the previously proposed role of the inhibitory Glu in obstructing correct engagement of the γ and β phosphates of ATP.

Enzyme Activity of HYPE Variants and Possible Protein Targets

The structures of the HYPE variants with ATP and ADP, direct binding measurements, and earlier work suggest that HYPE can function in protein AMPylation. However, the basal activity of the wild-type HYPE is probably lower when compared to the E234G variant. It has been previously documented that the proposed intramolecular inhibition in several FIC domains (Engel et al., 2012) can be reversed by replacing the critical inhibitory Glu residue by Gly, using autoAMPylation as a readout (Goepfert et al., 2013). Regardless of the physiological relevance of this replacement, conservation of the reaction mechanism (Garcia-Pino et al., 2014) would allow application of this strategy for the analysis of structure-function relationship. Using a chemo-enzymatic tagging and a Yn-6-ATP in vitro probe (Figure S7) (Heal et al., 2012) (compatible with the AMPylation reaction, see Grammel et al., 2011), we were able to show by fast in-gel fluorescence readout, that HYPE can autoAMPylate (Figure 7A). Furthermore, basal activity of the wild-type HYPE was drastically boosted by the E234G mutation. In contrast, mutations of the catalytic His363 or Mg^{2+}-coordinating Asp367 (in the context

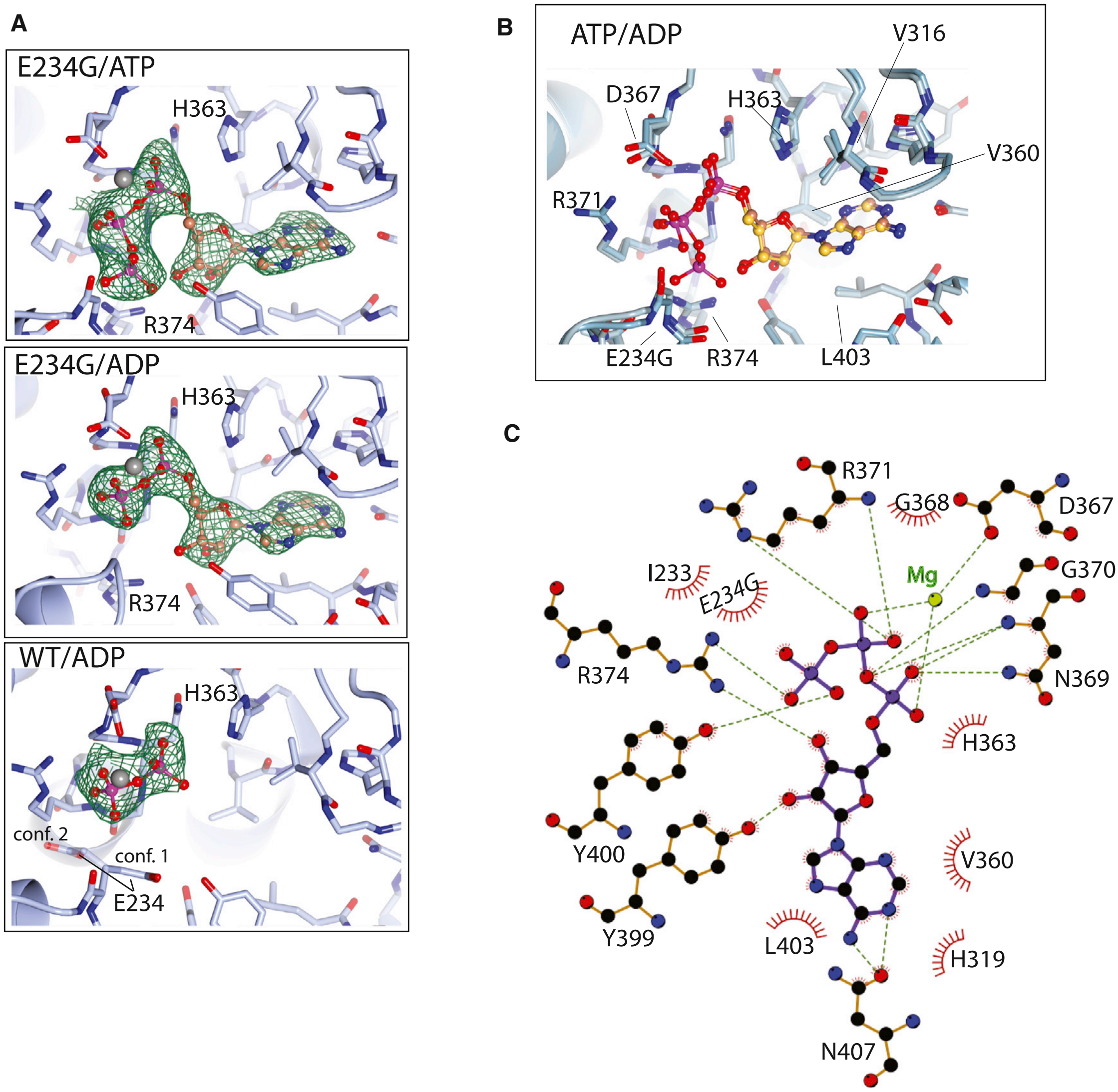

Figure 5. Structures of HYPE Variants with Bound ADP and ATP

(A) Binding pocket for ATP and ADP in E234G and wild-type (WT) variants; density of the cofactors, catalytic His 363, and γ-phosphate coordinating Arg 374 are indicated. For WT/ADP, two conformations (more abundant conf. 1 and conf. 2) of the side chain Glu 234 are indicated. Electron density around the adenosine part of the ligand was poor, and so it is not shown for clarity.

(B) Overlay of ATP and ADP in the binding pocket of E234G variant; some of the key residues are labeled. See also Figure S6.

(C) Coordination of ATP by E234G variant, represented as a LigPlot^{+} diagram.

of the E234G variant) abolished the enzyme activity (both values were about 4%–6% of control), consistent with the conserved reaction mechanism.

We used this autoAMPylation assay to test the functional implications of interdomain interactions on enzyme activity of the FIC domain. Removal of the TPR-motifs (residues 103–172) maintained a stable protein; however, further deletions from the N terminus up to residue 215, that removed the α helix linker, resulted in a highly unstable protein that was insoluble as a single entity. A comparison of constructs that incorporate (residues 103α434, E234G) and lack TPR-motifs (residues 172α445, E234G) show a clear reduction in autoAMPylation in the absence of TPR-motifs (Figure 7B). We also excluded the possibility that this reduction is due to the removal of sites of autoAMPylation that could be present in TPR-motifs. Using a fusion protein of the HYPE variant that is catalytically inactive (SUMO-tag, H363A), the AMPylation of this protein was also reduced to less than 10% when using an E234G construct lacking TPR-motifs. Furthermore, the replacement of potential AMPylation sites in the TPR-motifs (T168A, S170A, and Y172F) did not have an effect on the overall level of autoAMPylation (values within ± 10% of control). Together with structures of the HYPE constructs, this analysis of enzyme activity shows that the FIC domain interactions with the α helix linker and TPR-motifs are required for protein stability and efficient catalytic activity of the FIC domain.

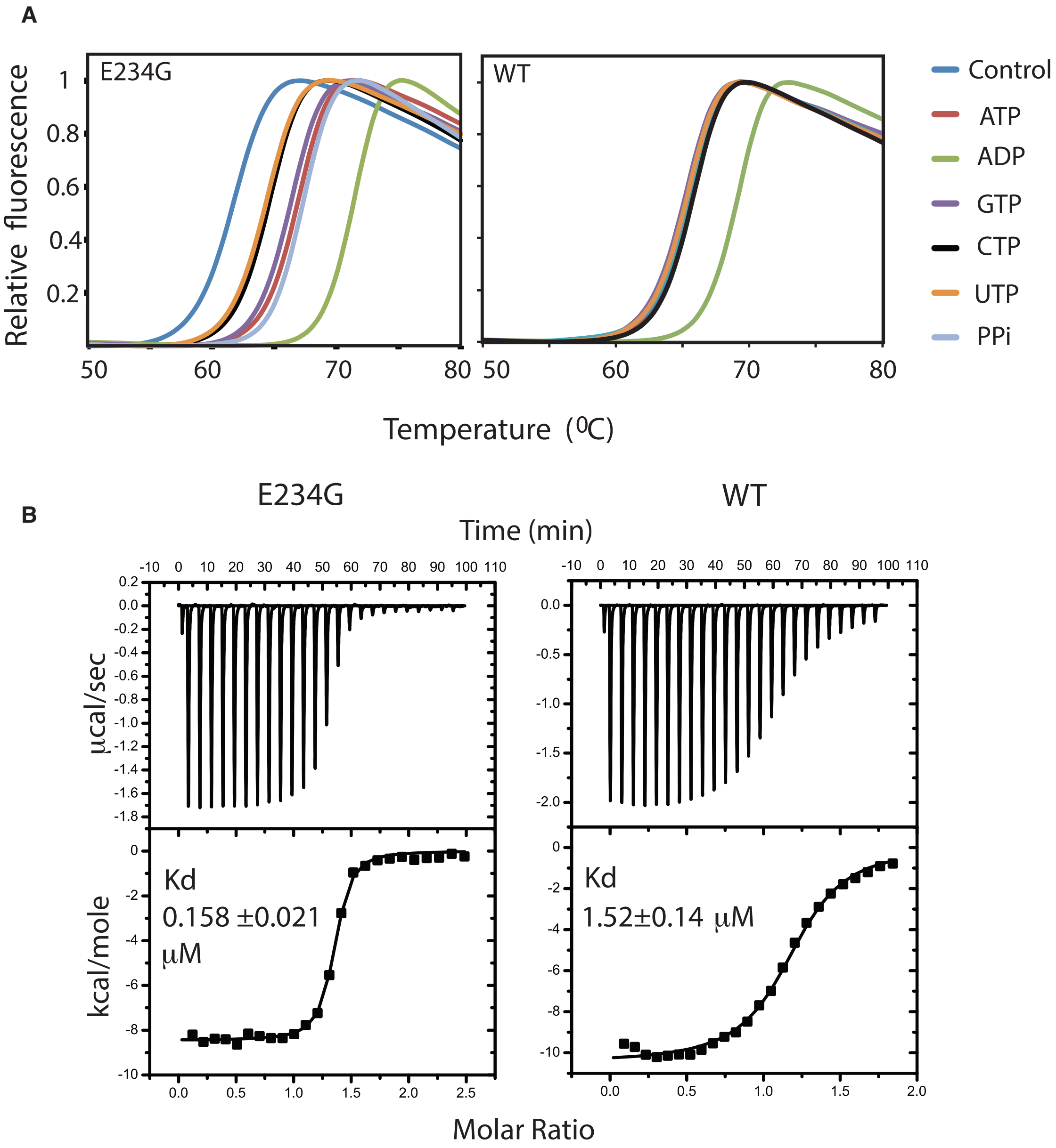

Figure 6. Cofactor Binding

(A) Differential scanning fluorimetry (thermal shift) analysis of HYPE E234G (left) and WT (right) in the absence (control) and presence of indicated compounds. The data are representative for two independent experiments with the same relative difference in Tm.

(B) Binding of ADP to the E234G (E234G, residues 103–445) (left) and WT variant (WT, residues 103–445) (right) was measured by ITC. The indicated errors represent the error of the fit.

AutoAMPylation activity of the FIC domain appears also to be affected by replacement of residues involved in dimerization or in the vicinity of the dimerization surface (Figure 7C). The activity of the purified L258D variant, which is a monomer in solution, was greatly reduced. Interestingly, some other mutations close to the dimerization surface, not sufficient to disrupt dimerization, also had an impact on autoAMPylation (Figure 7C, top). This suggests that the reduced enzyme activity of the L258D variant may not simply result from the generation of a monomeric form of HYPE. Additionally, the impact of the L258D mutation on autoAMPylation of the E234G, full-length variant, was observed in a cellular setting (Figure 7C, bottom).

While the measurements of autoAMPylation provide some insights into structure-function relationships, the physiological significance of autoAMPylation remains unclear. Furthermore, a cellular substrate of HYPE has not been defined (Garcia-Pino et al., 2014). In our initial experiments addressing this question, we used a Yn-6-ATP-based assay in vitro to compare proteins in cell extract that become AMPylated by HYPE, and by one of the well-studied FIC domain proteins VopS from *Vibrio*

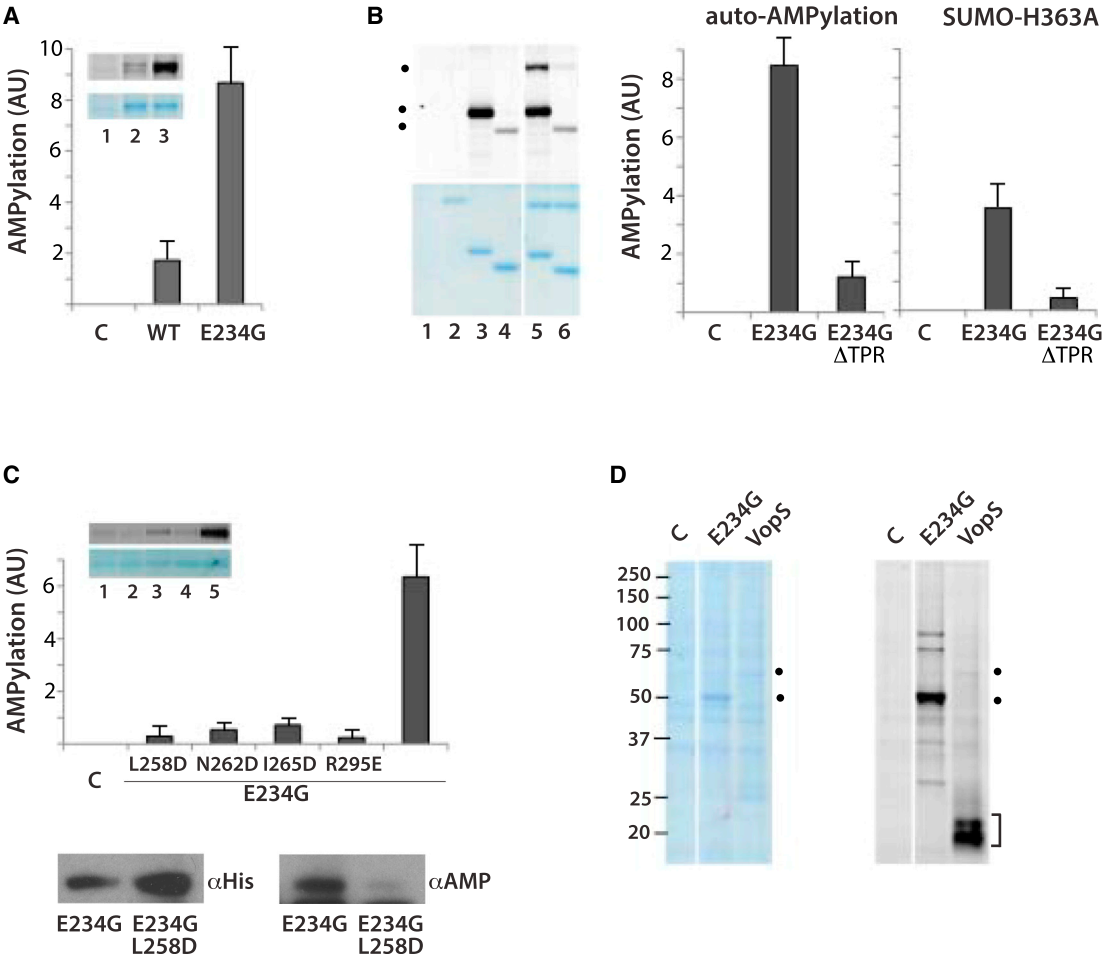

Figure 7. AMPylation Activity of HYPE

(A) Effect of E234G mutation on autoAMPylation of HYPE was analyzed by Yn-6-ATP-based assay, using WT and E234G variant (E234G) of HYPE (residues 103–434). For the control lane (C), HYPE protein was not included. Inset shows protein bands resolved by SDS-PAGE and Coomassie stained (bottom) and corresponding in-gel fluorescence (top). Fluorescence corresponding to autoAMPylation was quantified using ImageJ.

(B) Effect of deletion of TPR-motifs on AMPylation activity of HYPE was analyzed as in (A), using E234G (E234G, residues103–445) and E234G/ΔTPR (E234G, residues 172–445) variants of HYPE. In addition to autoAMPylation, AMPylation of H363A HYPE, containing SUMO-tag (SUMO-H363A, residues 103–445) was also analyzed. Left panel shows protein bands (bottom) and corresponding fluorescence (top). Lanes 1–6 correspond to; control without enzyme (1), SUMO-H363A (2), E234G (3), E324G/ΔTPR (4), E234G with SUMO-H363A (5), and E234G/ΔTPR with SUMO-H363A (6). Positions of AMPylated bands are indicated (.).

(C) Top panel shows effect of L258D, N262D, I265D, and R295E mutations on autoAMPylation of E234G HYPE (E234G, residues 172–445), analyzed as described in (A); inset shows protein bands (bottom) and corresponding fluorescence (top). Bottom panels show western blots of the full-length HYPE variants E234A and E234A/L258A, expressed in HEK293T cells, performed using either anti-His tag (αHis) (left) or anti-AMPThr (αAMP) antibodies (right).

(D) AMPylation of cellular proteins in vitro was performed using purified HYPE E234G (E234G, residues 103–445) and VopS in the presence of the cell lysate from HeLa cells. The cell lysate without added enzyme was used as a control (C). Left panel shows protein bands and the right panel corresponding fluorescence; autoAMPylation of HYPE E234G and VopS are indicated (.), as well as AMPylation in the area corresponding to mobility of small GTPases (]).

The indicated error bars in (A)–(C) represent SD from two experiments. See also Figure S7.

parahaemolyticus that lacks autoinhibition (Figure 7D). As expected, the main AMPylation targets of VopS are in agreement with molecular sizes corresponding to Rho family GTPases. In contrast, small GTPases did not seem to be targeted by E234G (or wild-type) HYPE and, in addition to strong autoAMPylation, several other proteins appear to be AMPylated by the E234G variant. These findings suggest that HYPE may have a range of interacting proteins and targets in cells, and that further development of cell permeable cofactor analogs allowing for profiling in live cells could provide a suitable route to identifying targets of HYPE presented in its physiologically relevant subcellular localization.

DISCUSSION

Recent findings that protein AMPylation is a novel PTM occurring in eukaryotic cells, highlighted the need to better define its role beyond bacterial infection, where FIC-domain containing enzymes are of bacterial origin (Yarbrough and Orth, 2009). We here describe the structural properties of HYPE; a potential

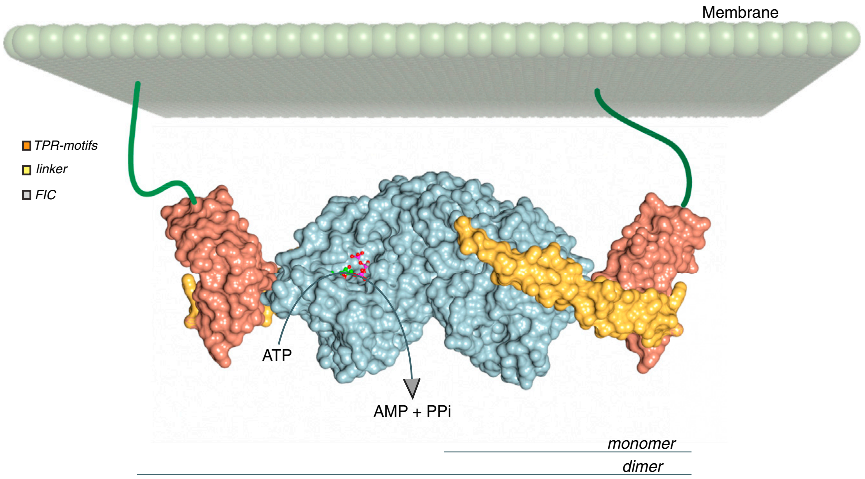

Figure 8. A Model Depicting HYPE Dimers in the Membrane Vicinity

Properties of HYPE structure are summarized by surface representation of a dimer. A HYPE dimer is featured relative to the plane of a cellular membrane, taking into consideration that the 20 amino acid long transmembrane domains would be imbedded into the membrane and that flexible (largely unstructured) linkers of about 60 residues connect transmembrane domains and TPR-motifs.

AMPylator encoded by eukaryotic genomes, and further analyze and discuss the functional implications of its distinct features.

Eukaryotic HYPE proteins are membrane proteins that uniquely combine one transmembrane helix, two TPR-motifs, and the FIC domain. The structure of the large portion of this protein, including the TPR-motifs, linker region, and FIC domain, summarized in Figure 8, shows that intramolecular interactions result in a rigid arrangement that is extended further by dimerization. TPR-motifs are positioned so that the active site opening in the FIC domain is exposed. Also, intramolecular interactions leave TPR-motifs free to engage in other protein-protein interactions. There are two TPR-motifs of each monomer that are placed at the opposite side of the dimer surface, precluding formation of a larger platform of TPR repeats.

The overall structure of HYPE shows some distinct properties when compared to other proteins that harbor FIC domains or with proteins where TPR domains are combined with other enzyme activities. It appears that to date, FIC-mediated dimerization has not been observed for bacterial effectors, which are in most cases monomeric in solution (Garcia-Pino et al., 2014). An interaction of the FIC domain with a defined domain structure has been described only in one other protein, AnkX, where a similar repeat domain, ankyrin repeat, interacts with the FIC domain so that surfaces usually involved in interactions with other binding partners are involved in intramolecular interactions (Campanacci et al., 2013); this is clearly not the case for HYPE (Figures 1 and S3). The relative orientation of the FIC domain and TPR-motifs in HYPE is also distinct from those observed in PP5 (Figure S3) or Cyp40, two examples where the structures for the full-length TPR-proteins have been determined (Allan and Ratajczak, 2011). In PP5, the TPR domain engages with the catalytic channel of the phosphatase domain, restricting access to the catalytic site, while in Cyp40 the two domains (TPR domain and peptidyl prolyl isomerase domain) are completely independent (Taylor et al., 2001; Yang et al., 2005). Positions observed in HYPE are intermediate between these two examples; there are clear interaction surfaces, centered on the C-terminal α helix of the TPR domain, leaving both the FIC active site and grooves of TPR-motifs exposed (Figures 1 and S3).

TPR repeats (usually three or more) have been predicted to occur in a large number of proteins (about 5,000), and many have been suggested to act as scaffolds for the assembly of multiprotein complexes, such as protein folding complexes, anaphase promoting complex, or the peroxisomal import receptor complex (Allan and Ratajczak, 2011; Blatch and Lässle, 1999; D'Andrea and Regan, 2003; Zeytuni and Zarivach, 2012). In addition to adaptor proteins (such as Hop), some TPR proteins that contain enzyme domains (including PP5 and Cyp40) have been suggested to be part of larger complexes. Although this remains an interesting possibility for HYPE, information available for its function is limited, and there is no clear evidence that it is incorporated into larger complexes. Another possibility is that the TPR-motifs have a role in the recognition of a protein target. TPR-motifs present in the α subunit of tetrameric ($\alpha2\beta2$) propyl P4H are mainly involved in substrate (collagen) recognition and have an essential role in collagen synthesis (Pekkala et al., 2004). Furthermore, among the FIC domain proteins, the target binding sites are not conserved and each recognition site is largely influenced by unique target-recognition elements, additional to the FIC α-helical core. For example, in IbpAFic2, an additional "arm domain" provides a large set of unique contacts with the target. This is also an all α-helical lobe domain that protrudes N-terminally from the FIC domain (Xiao et al., 2010). Interestingly, the relative orientation of the arm domain and the FIC core in IbpAFic2 is such that the active site is exposed, as observed for HYPE (Figure S3).

So far, the best-defined targets for FIC–containing proteins are GTPases: Rho GTPases are AMPylated by VopS and IbpA; Rab1 and Rab35 are phosphocholinated by AnkX; and EF-Tu is phosphorylated by Doc; a different example is provided by AvrAC, that targets two kinases (BIK1 and RIPK) by UMPylation (Garcia-Pino et al., 2014). In all these cases, substrate recognition is very specific, and the effect on target function is inhibitory. However, these are all bacterial effectors and targets, and the function of HYPE could be different. Our initial data show clear differences between VopS recognition of Rho GTPases and distinct, potential substrate targets of HYPE (Figure 7D). Assuming the involvement of TPR-motifs in substrate recognition, it has been documented that these motifs have a range of interaction partners. Among TPR-motifs, only the TPRs from p67phox bind a GTPase, namely Rac. The recognition surface is, however, not placed in the TPR groove, but, instead, the binding occurs almost exclusively to the β hairpin insertion element, unique to p67phox (Lapouge et al., 2000).

Our data describing the cofactor binding, where binding of ATP to wild-type HYPE is obstructed by the conserved glutamic

acid residue (Figure 5), raise two possibilities. An explanation is based on a concept that the inhibitory α helix and the critical glutamic acid residue obstructing binding of the γ-phosphate of ATP can be removed in the course of a physiologically relevant activation process. This argument is based on a comprehensive analysis of FIC-domain proteins that has identified the conserved inhibitory motif and its coevolution with the conserved FIC motif and putative AMPylation function (Engel et al., 2012). The cofactor binding pockets of FIC domain proteins DOC and AnkX involved in phosphorylation and phosphocholination, respectively, are clearly different from an ADP or ATP binding site in HYPE, and several other proteins with the canonical FIC motif (Engel et al., 2012; Goepfert et al., 2013; Palanivelu et al., 2011). Cofactors are placed in a different orientation, and elements specific for DOC or AnkX facilitate their binding and/or preclude orientation observed in HYPE (Campanacci et al., 2013; Castro-Roa et al., 2013). It has also been observed that wild-type HYPE has low basal levels of AMPylation that are enhanced rather than created by the E234G replacement (Worby et al., 2009; Mattoo et al., 2011; Engel et al., 2012; Yu et al., 2014). However, one important unresolved question with respect to autoinhibition is related to the mechanism that would overcome this inhibitory constraint, leading to activation. The structure of HYPE shows that αinh is held in place by the rest of the structure, and weakening the interaction of αinh with the FIC active site could require significant conformational changes to move the glutamate away from the active site. Therefore, an explanation that the glutamate discriminating against ATP evolved to preclude ATP binding in favor of another cofactor (Campanacci et al., 2013) provides a plausible alternative. While further analysis of a physiologically relevant new cofactor for HYPE would require other experimental approaches, binding studies shown here (Figure 6) suggest that compounds related to nucleotide bisphosphates could be relevant candidates.

Unlike most other FIC domain proteins, HYPE contains a transmembrane helix and is expected to function in membrane proximity. A recent study on *Drosophila* has suggested that HYPE is localized at the endoplasmic reticulum rather than the plasma membrane (Rahman et al., 2012); our initial data for localization of human HYPE in transfected cells are consistent with this finding (data not shown). Because of the dimer structure of HYPE, and, consequently, two anchor points with cellular membranes per dimer, it is likely that the positioning with respect to the membrane could be less flexible, with restricted orientation (Figure 8). This, in turn, could influence protein-protein interactions and contribute to a more precise assembly of potential, larger complexes or influence target selection.

New insights obtained by structural and functional characterization of HYPE, together with methodologies that may allow discovery of protein targets in cells, provide important steps toward further elucidation of physiological roles of HYPE and the significance of posttranslational modifications mediated by this unique eukaryotic protein.

EXPERIMENTAL PROCEDURES

Constructs

Full length constructs for mammalian expression were cloned into pcDNA-Dest40 (Life Technologies) in frame with a C-terminal mVENUS fluorescent tag and a HIS-tag. Constructs for bacterial expression were cloned into pOPINS (Oxford Protein Production Facility) with boundaries 103–434, 103–445, and 172–445. Point mutations E234G, H363A, T168A, S170A, Y172F, L258D, N262D, I265D, R295E, E259G, E263G, and D367G were introduced in constructs for bacterial expression or, when specified, also in mammalian expression vector. Combinations of point mutations are indicated for specific constructs.

Expression and Purification

The *E. coli* strain C41(DE3) (Lucigen) was used for expression of most constructs. Transformed cells were grown in 2×YT media containing 50 μg/ml kanamycin at 37°C until an optical density$_{600}$ of between 0.5 and 0.8. The temperature was decreased to 20°C and expression was induced through the addition of 0.1 mM isopropyl β-D-1-thiogalactopyranoside for 16 hr. Cell lysis was performed on frozen pellets using 25 mM Tris.Cl, 250 mM NaCl, 40 mM Imidazole, and 5 mM Benzamidine.HCl, pH 8.0 with the addition of 0.1 mg/ml lysozyme and 1% (v/v) Triton X-100 at 4°C. Purification was performed using Ni^{2+} chelating chromatography, followed by cleavage of the His-SUMO tag through the addition of Ulp1 protease. Subsequently, Q-sepharose ion-exchange chromatography and gel filtration chromatography were performed, the protein concentrated to 20 mg/ml and aliquots snap frozen in liquid N_2, and stored at −80°C. Transfection of full-length constructs into Freestyle human embryonic kidney (HEK)293F mammalian cells was performed according to manufacturers instructions (Life Technologies).

Crystallography

Apo, wild-type HYPE, was concentrated to 9 mg/ml prior to crystallization via vapor diffusion with 20% polyethylene glycol 3,350, 200 mM Na K Tartrate, and 100mM Bis-Tris Propane pH 7.5 as mother liquor mixed in a 2:1 ratio, plates were then stored at 16°C for crystal growth. Mutant E234G HYPE was crystallized in the same condition. Mutant crystals of a higher quality were achieved with the addition of 10 mM Yn6ATP analog. Initial diffraction experiments revealed no analog in the active site, however. Following crystallization, all crystals were soaked in a cryoprotectant containing mother liquor with a 50% saturated solution of sucrose. Mutant crystals were also soaked with 10mM ATP or ADP alongside 10mM $MgCl_2$.

Data were collected using synchrotron radiation from Diamond Light Source; using line scans to maximize data collection time for apo and APCPP data sets. The ATP data set was collected at Synchrotron Soleil and the ADP data set using a Rigaku home source.

All data sets were processed using the integration program XDS (Kabsch, 2010) with further processing using the CCP4 program Aimless (Winn et al., 2011). Molecular replacement was carried out using the program PHASER (McCoy et al., 2007; Winn et al., 2011). The search model was created from the FIC domain structure 3CUC from the Protein Data Bank (PDB), from *Bacterioedes thetaiotaomicron,* paired back to shared atoms using the program CHAINSAW (Stein, 2008; Winn et al., 2011). There were two molecules that were found which were rebuilt using the program COOT (Emsley et al., 2010; Winn et al., 2011). N-terminal TPR-motifs were initially not readily visible. To search for the expected helical domains, real space searching was applied with the program FFEAR (Cowtan, 1998; Winn et al., 2011) using a nine residues polyalanine helix as the search model. This resulted in multiple hits together close to the two FIC domains. Careful rebuilding and extension of this region with subsequent refinement using the BUSTER program (Bricogne et al., 2011) produced the long linker helix followed by the N-terminal TPR-motifs. Finally, water molecules and ligands were built into the structure, and the final structure was validated using the Molprobity server (Chen et al., 2010). LigPlot+ (Laskowski and Swindells, 2011) was used for generation of ligand binding diagrams.

ITC

ITC measurements were performed as described previously (Bunney et al., 2009, 2012). Heats of interaction were measured on a VP-ITC system (Microcal) with a cell volume of 1.458 ml. HYPE molecules were dialyzed for 16 hr in ITC buffer (25 mM Tris.Cl, 150 mM NaCl, 50 mM $MgCl_2$, and 1 mM TCEP, pH 8.0). HYPE was loaded in the sample cell at 100 μM and titrated with ADP in the syringe (1 mM). The titrations were performed while samples were being stirred at 260 revolutions per minute at 20°C. A total of 25 injections were carried out,

with 10 μl injected each time (except the first injection, when 3 μl was injected), and a 4 min interval between each injection to allow the baseline to stabilize. The data were fitted with a single site model to calculate the number of binding sites (n), the binding constant (Ka), the change in enthalpy (ΔHo), and change in entropy (ΔS) using Origin software (Microcal, 2004).

Differential Scanning Fluorimetry

Ligand binding experiments were performed on a Stratagene MX3005P machine (Agilent Technologies) running MxPro qPCR software (Agilent Technologies, 2009). Experiments were carried out in 96-well real-time PCR plates with 20 μl samples (duplicates) containing 2 μM protein, 200 μM ligand (or vehicle), 5 mM $MgCl_2$, and 10 × SYPRO Orange dye in 25 mM Tris.Cl, 150 mM NaCl, and 1 mM TCEP, pH 8.0. Fluorescence of the SYPRO Orange dye was monitored in each well over 25–95°C. Curves were analyzed in MS Excel (Microsoft, 2011).

Additional experimental procedures describing western blotting of full-length HYPE constructs expressed in HEK293F cells, SAXS, and AMPylation assays are included in Supplemental Experimental Procedures.

ACCESSION NUMBERS

The crystal structures of HYPE have been deposited in the PDB with the accession codes, 4U04 (apo), 4U07 (E234G variant/ATP bound), 4U0S (E234G variant/ATD bound), 4U0U (wild type/ATD bound), and 4U0Z (E234G variant/APCPP bound).

SUPPLEMENTAL INFORMATION

Supplemental Information includes Supplemental Experimental Procedures and seven figures and can be found with this article online at http://dx.doi.org/10.1016/j.str.2014.10.007.

AUTHOR CONTRIBUTIONS

M.K., E.W.T., and T.D.B. planned the project. T.D.B. and A.R.C. designed and performed experiments covering protein purification (T.D.B.), biophysical characterization (T.D.B.), and crystallography (A.R.C.) and analyzed the data. M.B. designed and performed experiments for AMPylation and thermal shift assays and analyzed the data. D.E. analyzed data from SAXS measurements. A.R.C., T.D.B., and M.K. prepared the figures. M.K. wrote the manuscript and all authors read, corrected, and approved the final manuscript.

ACKNOWLEDGMENTS

We thank Catherine S. Watson for assistance with crystallography and ITC experiments and Alice Clark for help with SAXS measurements. We also acknowledge support from the synchrotrons Diamond, Soleil, and European Synchrotron Radiation Facility (ESRF). The M.K. laboratory is supported by Cancer Research UK and M.B. by German Research Foundation (DFG, grant BR 4387/1-1) and European Union (Marie Curie Intra European Fellowship PIEF-GA-2011-299740).

Received: July 16, 2014
Revised: September 22, 2014
Accepted: October 6, 2014
Published: November 26, 2014

REFERENCES

Allan, R.K., and Ratajczak, T. (2011). Versatile TPR domains accommodate different modes of target protein recognition and function. Cell Stress Chaperones *16*, 353–367.

Blatch, G.L., and Lässle, M. (1999). The tetratricopeptide repeat: a structural motif mediating protein-protein interactions. BioEssays *21*, 932–939.

Bricogne, G., Blanc, E., Brandl, M., Flensburg, C., Keller, P., Paciorek, W., Roversi, P., Sharff, A., Smart, O.S., and Vonrhein, C. (2011). BUSTER Version 2.10.0. (Cambridge, UK: Global Phasing Ltd.).

Broncel, M., Serwa, R.A., and Tate, E.W. (2012). A new chemical handle for protein AMPylation at the host-pathogen interface. ChemBioChem *13*, 183–185.

Bunney, T.D., Opaleye, O., Roe, S.M., Vatter, P., Baxendale, R.W., Walliser, C., Everett, K.L., Josephs, M.B., Christow, C., Rodrigues-Lima, F., et al. (2009). Structural insights into formation of an active signaling complex between Rac and phospholipase C gamma 2. Mol. Cell *34*, 223–233.

Bunney, T.D., Esposito, D., Mas-Droux, C., Lamber, E., Baxendale, R.W., Martins, M., Cole, A., Svergun, D., Driscoll, P.C., and Katan, M. (2012). Structural and functional integration of the PLCγ interaction domains critical for regulatory mechanisms and signaling deregulation. Structure *20*, 2062–2075.

Campanacci, V., Mukherjee, S., Roy, C.R., and Cherfils, J. (2013). Structure of the Legionella effector AnkX reveals the mechanism of phosphocholine transfer by the FIC domain. EMBO J. *32*, 1469–1477.

Castro-Roa, D., Garcia-Pino, A., De Gieter, S., van Nuland, N.A., Loris, R., and Zenkin, N. (2013). The Fic protein Doc uses an inverted substrate to phosphorylate and inactivate EF-Tu. Nat. Chem. Biol. *9*, 811–817.

Chen, V.B., Arendall, W.B., 3rd, Headd, J.J., Keedy, D.A., Immormino, R.M., Kapral, G.J., Murray, L.W., Richardson, J.S., and Richardson, D.C. (2010). MolProbity: all-atom structure validation for macromolecular crystallography. Acta Crystallogr. D Biol. Crystallogr. *66*, 12–21.

Cowtan, K. (1998). Modified phased translation functions and their application to molecular-fragment location. Acta Crystallogr. D Biol. Crystallogr. *54*, 750–756.

Cruz, J.W., Rothenbacher, F.P., Maehigashi, T., Lane, W.S., Dunham, C.M., and Woychik, N.A. (2014). Doc toxin is a kinase that inactivates elongation factor Tu. J. Biol. Chem. *289*, 7788–7798.

D'Andrea, L.D., and Regan, L. (2003). TPR proteins: the versatile helix. Trends Biochem. Sci. *28*, 655–662.

Deribe, Y.L., Pawson, T., and Dikic, I. (2010). Post-translational modifications in signal integration. Nat. Struct. Mol. Biol. *17*, 666–672.

Emsley, P., Lohkamp, B., Scott, W.G., and Cowtan, K. (2010). Features and development of Coot. Acta Crystallogr. D Biol. Crystallogr. *66*, 486–501.

Engel, P., Goepfert, A., Stanger, F.V., Harms, A., Schmidt, A., Schirmer, T., and Dehio, C. (2012). Adenylylation control by intra- or intermolecular active-site obstruction in Fic proteins. Nature *482*, 107–110.

Feng, F., Yang, F., Rong, W., Wu, X., Zhang, J., Chen, S., He, C., and Zhou, J.M. (2012). A Xanthomonas uridine 5′-monophosphate transferase inhibits plant immune kinases. Nature *485*, 114–118.

Finn, R.D., Mistry, J., Tate, J., Coggill, P., Heger, A., Pollington, J.E., Gavin, O.L., Gunasekaran, P., Ceric, G., Forslund, K., et al. (2010). The Pfam protein families database. Nucleic Acids Res. *38*, D211–D222.

Garcia-Pino, A., Zenkin, N., and Loris, R. (2014). The many faces of Fic: structural and functional aspects of Fic enzymes. Trends Biochem. Sci. *39*, 121–129.

Goepfert, A., Stanger, F.V., Dehio, C., and Schirmer, T. (2013). Conserved inhibitory mechanism and competent ATP binding mode for adenylyltransferases with Fic fold. PLoS ONE *8*, e64901.

Grammel, M., Luong, P., Orth, K., and Hang, H.C. (2011). A chemical reporter for protein AMPylation. J. Am. Chem. Soc. *133*, 17103–17105.

Ham, H., and Orth, K. (2011). De-AMPylation unmasked: modulation of host membrane trafficking. Sci. Signal. *4*, pe42.

Heal, W.P., Wright, M.H., Thinon, E., and Tate, E.W. (2012). Multifunctional protein labeling via enzymatic N-terminal tagging and elaboration by click chemistry. Nat. Protoc. *7*, 105–117.

Kabsch, W. (2010). Xds. Acta Crystallogr. D Biol. Crystallogr. *66*, 125–132.

Kamath, K.S., Vasavada, M.S., and Srivastava, S. (2011). Proteomic databases and tools to decipher post-translational modifications. J. Proteomics *75*, 127–144.

Kinch, L.N., Yarbrough, M.L., Orth, K., and Grishin, N.V. (2009). Fido, a novel AMPylation domain common to fic, doc, and AvrB. PLoS ONE *4*, e5818.

Lapouge, K., Smith, S.J., Walker, P.A., Gamblin, S.J., Smerdon, S.J., and Rittinger, K. (2000). Structure of the TPR domain of p67phox in complex with Rac.GTP. Mol. Cell *6*, 899–907.

Laskowski, R.A., and Swindells, M.B. (2011). LigPlot+: multiple ligand-protein interaction diagrams for drug discovery. J. Chem. Inf. Model. *51*, 2778–2786.

Lewallen, D.M., Sreelatha, A., Dharmarajan, V., Madoux, F., Chase, P., Griffin, P.R., Orth, K., Hodder, P., and Thompson, P.R. (2014). Inhibiting AMPylation: a novel screen to identify the first small molecule inhibitors of protein AMPylation. ACS Chem. Biol. *9*, 433–442.

Mattoo, S., Durrant, E., Chen, M.J., Xiao, J., Lazar, C.S., Manning, G., Dixon, J.E., and Worby, C.A. (2011). Comparative analysis of Histophilus somni immunoglobulin-binding protein A (IbpA) with other fic domain-containing enzymes reveals differences in substrate and nucleotide specificities. J. Biol. Chem. *286*, 32834–32842.

McCoy, A.J., Grosse-Kunstleve, R.W., Adams, P.D., Winn, M.D., Storoni, L.C., and Read, R.J. (2007). Phaser crystallographic software. J. Appl. Cryst. *40*, 658–674.

Müller, M.P., Peters, H., Blümer, J., Blankenfeldt, W., Goody, R.S., and Itzen, A. (2010). The Legionella effector protein DrrA AMPylates the membrane traffic regulator Rab1b. Science *329*, 946–949.

Palanivelu, D.V., Goepfert, A., Meury, M., Guye, P., Dehio, C., and Schirmer, T. (2011). Fic domain-catalyzed adenylylation: insight provided by the structural analysis of the type IV secretion system effector BepA. Protein Sci. *20*, 492–499.

Pekkala, M., Hieta, R., Bergmann, U., Kivirikko, K.I., Wierenga, R.K., and Myllyharju, J. (2004). The peptide-substrate-binding domain of collagen prolyl 4-hydroxylases is a tetratricopeptide repeat domain with functional aromatic residues. J. Biol. Chem. *279*, 52255–52261.

Rahman, M., Ham, H., Liu, X., Sugiura, Y., Orth, K., and Krämer, H. (2012). Visual neurotransmission in Drosophila requires expression of Fic in glial capitate projections. Nat. Neurosci. *15*, 871–875.

Roy, C.R., and Mukherjee, S. (2009). Bacterial FIC proteins AMP up infection. Sci. Signal. *2*, pe14.

Stein, N. (2008). CHAINSAW: a program for mutating pdb files used as templates in molecular replacement. J. Appl. Cryst. *41*, 641–643.

Taylor, P., Dornan, J., Carrello, A., Minchin, R.F., Ratajczak, T., and Walkinshaw, M.D. (2001). Two structures of cyclophilin 40: folding and fidelity in the TPR domains. Structure *9*, 431–438.

Winn, M.D., Ballard, C.C., Cowtan, K.D., Dodson, E.J., Emsley, P., Evans, P.R., Keegan, R.M., Krissinel, E.B., Leslie, A.G., McCoy, A., et al. (2011). Overview of the CCP4 suite and current developments. Acta Crystallogr. D Biol. Crystallogr. *67*, 235–242.

Worby, C.A., Mattoo, S., Kruger, R.P., Corbeil, L.B., Koller, A., Mendez, J.C., Zekarias, B., Lazar, C., and Dixon, J.E. (2009). The fic domain: regulation of cell signaling by adenylylation. Mol. Cell *34*, 93–103.

Xiao, J., Worby, C.A., Mattoo, S., Sankaran, B., and Dixon, J.E. (2010). Structural basis of Fic-mediated adenylylation. Nat. Struct. Mol. Biol. *17*, 1004–1010.

Yang, J., Roe, S.M., Cliff, M.J., Williams, M.A., Ladbury, J.E., Cohen, P.T., and Barford, D. (2005). Molecular basis for TPR domain-mediated regulation of protein phosphatase 5. EMBO J. *24*, 1–10.

Yarbrough, M.L., and Orth, K. (2009). AMPylation is a new post-translational modiFICation. Nat. Chem. Biol. *5*, 378–379.

Yarbrough, M.L., Li, Y., Kinch, L.N., Grishin, N.V., Ball, H.L., and Orth, K. (2009). AMPylation of Rho GTPases by Vibrio VopS disrupts effector binding and downstream signaling. Science *323*, 269–272.

Yu, X., Woolery, A.R., Luong, P., Hao, Y.H., Grammelc, M., Westcottc, N., Parka, J., Wanga, J., Biana, X., Demirkana, G., Hang, H.C., et al. (2014). Click chemistry-based detection of global pathogen-host AMPylation on self-assembled human protein microarrays. Mol Cell Proteomics, Jul 29. pii: mcp.M114.041103. [Epub ahead of print].

Zeytuni, N., and Zarivach, R. (2012). Structural and functional discussion of the tetra-trico-peptide repeat, a protein interaction module. Structure *20*, 397–405.

Structure, Volume *22*

Supplemental Information

Crystal Structure of the Human, FIC-Domain Containing Protein HYPE and Implications for Its Functions

Tom D. Bunney, Ambrose R. Cole, Malgorzata Broncel, Diego Esposito, Edward W. Tate, and Matilda Katan

```
[Caenorhabditis     MSVRRRT---HSDDFSYL-------------LEKTRRPSK-----LN----VVQEDPKSA
[Drosophila         ----MGT---EAEQPSPPAQQQDQEN---PPLCKAQNPKP--------------------
[Xenopus            ---------------------------------------------------MAVTECEWA
[Gallus             MAERGGRGREEAGQPGWSRQQEAEGGGQGRGSTKGDVPRIRAGSRMNLISMATEPELKWV
[Mus                ------------------------------------------MILMPMASVVAVAEPKWV
[Homo               ------------------------------------------MMLIPMASVMAVTEPKWV

[Caenorhabditis     PPQGYSLTTVIIISVLVSLICQHFVPYAVST--LHTVIKNSPKQKSSPPPSNRLNIGFIS
[Drosophila         ----ARLYRFVLIFVAGSLAAWTF-HALSSTN-LVWKLRQLHHLPTAHYLQTRDEFALYS
[Xenopus            SLGSRIGLRAALVLLSGSLLVVLF-PLSGLEHQYRTALNIL------LQCN---LWGGDD
[Gallus             ALWVRVRWAAVLVLLLGSLLLLLL-PLPAVEEKCHAMLRGL------SFLRSKLGTGYTG
[Mus                SVWGRFLWMALLSMALGSLLALLL-PLGVVEEHCLAVLRGF------HLLRSKLDRAQLV
[Homo               SVWSRFLWVTLLSMVLGSLLALLL-PLGAVEEQCLAVLKGL------YLLRSKPDRAQHA
                               :     **    :             :.                 .

[Caenorhabditis     GNSPEKYAPAVQKPTFLVDPIYDEKWKGIQTAVPVMSTQ---TDEKRENDPAKVKEAILA
[Drosophila         VEE-------------------LNAFKEFYDKSVSDSVGAS----YTEAEQTNIKEALGA
[Xenopus            R----------------------HTFTGQTRGLAVASTAIELLVLKQKPTSDVKFEAKAA
[Gallus             V----------------------TRYTGQSTGLSVTSQGLELLVLKGKASPEVKLEAKAA
[Mus                V----------------------PKCTSLCTELSVSSRDAGLLTVKTTASPAGKLEAKAA
[Homo               A----------------------TKCTSPSTELSITSRGATLLVAKTKASPAGKLEARAA
                                              .         *                  **  *
                    TPR 1 α1        TPR 1 α2          TPR 2 α1          TPR 2 α2
[Caenorhabditis     AKAAGRSRKDGNLERAMTIMEHAMALAPTNPQILIEMGQIREMH-NELVEADQCYVKALA
[Drosophila         LRMAQDLYLAGKDDKAARLFEHALALAPRHPEVLLRYGEFLEHNQRNIVLADQYYFQALT
[Xenopus            LNQALEMKRQGKKEKAHKLLHHALKMDPDHVDALNELGILLEEE-KDIIQADYLYSKALT
[Gallus             LNQALEMKRQGKREKAHKLFVYALKMDPDYVDALNEFGIFSEEE-KDILQADYLYSKALT
[Mus                LNQALEMKRQGKRGKAHKLFLHALKMDPGFVDALNEFGIFSEED-KDIIQADYLYTRALT
[Homo               LNQALEMKRQGKREKAQKLFMHALKMDPDFVDALTEFGIFSEED-KDIIQADYLYTRALT
                     . *      *:  :*  :: :*: : *   : * . * : * . .::: **  * :**:
                                        Linker α                      FIC α inh
[Caenorhabditis     YDPGNSEALVLRARTTPLVSAIDRKMLRSVHDLRDEFNHL-QHSTALRRMMRETYFLYVY
[Drosophila         ISPSNSEALANRQRTADVVQSLDERRLESLDSKRDALSAIHESNGALRRAKKEAYFQHIY
[Xenopus            ISPHNEKALINRDRTLPLVEEIDQRYFSLIDSKVKKLMSIPKGNPALRRVMEESYYHHIY
[Gallus             LSPCNEKALINRDRTLPLVEEIDQRYFSIIDSKVKKVMAIPKGNSALRRVMEESYYHHIY
[Mus                ISPFHEKALVNRDRTLPLVEEIDQRYFSVIDSKVKKVMSIPKGSSALRRVMEETYYHHIY
[Homo               ISPYHEKALVNRDRTLPLVEEIDQRYFSIIDSKVKKVMSIPKGNSALRRVMEETYYHHIY
                     .* ..:**  * **  :*. :*.: :  :..  . .  : : . ****  .*:*: ::*
                                 FIC α pre A             FIC α pre B
[Caenorhabditis     HTVAIEGNTLSLGQTRAILESGMVIPGKSIREHNEVIGMDAALRFLNCSLLSKEHDEISI
[Drosophila         HSVGIEGNTMTLAQTRSILETRMAVDGKSIDEHNEILGMDLAMKYINASLVQK-ID-ITI
[Xenopus            HTVAIEGNTLSLSEIRHIIETRYAVPGKSLEEQNEVIGMHAAMKYVNATLVSR-IGSVTI
[Gallus             HTVAIEGNTLTLSEIRHIIETRYAVPGKSLVEQNEVIGMHAALKYVNTTLVSR-IGSVTI
[Mus                HTVAIEGNTLTLSEIRHILETRYAVPGKSLEEQNEVIGMHAAMKYINTTLVSR-IGSVTM
[Homo               HTVAIEGNTLTLSEIRHILETRYAVPGKSLEEQNEVIGMHAAMKYINTTLVSR-IGSVTI
                    *:*.*****::*.: * *:*:  .: ***: *:**::**. *::::* :*:.:    :::
                       FIC α1                   FIC - flap     FIC α2
[Caenorhabditis     DDILEMHRRVLGNADPVEAGRIRTTQVYVGRFTPVSPEYVMEQLKDIVDWLNDESTLTID
[Drosophila         KDILELHRRVLGHVDPIEGGEFRRNQVYVGGHIPPGPGDLALLMQRFERWLNSEHSSTLH
[Xenopus            DNILEIHRRILGYVDPVEAGRFRRNQVFVGHHIPPHPRDVEKLMQEFVQWLNSEEAMSLH
[Gallus             TDILEIHRRVLGYADPVEAGRFRTTQVFVGHHIPPHPQDVGKQMQEFVQWLNSEDAMSLH
[Mus                DDMLEIHRRVLGYVDPVEAGRFRRTQVLVGHHIPPHPRDVEKQMQEFTQWLNSEDAMNLH
[Homo               SDVLEIHRRVLGYVDPVEAGRFRTTQVLVGHHIPPHPQDVEKQMQEFVQWLNSEEAMNLH
                     ::**:***:** .**:*.*.:* .** ** . *  *  :   :: :  ***.* : .:.

                        FIC α3                FIC α4                 FIC α post A
[Caenorhabditis     PIERAAIAHYKLVLVHPFTDGNGRTARLLLNLIMMRSGFPPVILPVETRAEYYASLHVAN
[Drosophila         PVNYAALAHYKLVHIHPFVDGNGRTSRLLMNTLLMRAGYPPVIIPKQQRSKYYHFLKLAN
[Xenopus            PVEFAALAHYKLVYIHPFVDGNGRTSRLLMNLILMQAGYPPITVRKEQRSEYYHVLEIAN
[Gallus             PVEFAALAHYKLVYVHPFIDGNGRTSRLLMNLILMQAGYPPITIRKEQRAEYYHVLEVAN
[Mus                PVEFAALAHYKLVYIHPFIDGNGRTSRLLMNLILMQAGYPPITIRKEQRSEYYHVLEVAN
[Homo               PVEFAALAHYKLVYIHPFIDGNGRTSRLLMNLILMQAGYPPITIRKEQRSDYYHVLEAAN
                    *:: **:****** :*** ******:***:* ::*::*:**: :  : *:.**  *. **
                                    FIC α post B
[Caenorhabditis     LGDLRPFVRYVAKHSEASIQRYIGAMKTSSDNILNSGDSKLTPEESEVSEKIEAECRA--
[Drosophila         EGDIRPFVRFIADCTEKTLDLYLWATSDLPQQIPM-----LIQTESEAGERLAQMQSPNV
[Xenopus            EGDVRPFIRFIAKCTESTLDLLLIATAEHPVGLPE-----PNHGFSECKQTITIKT----
[Gallus             EGDVRPFIRFIAKCTETTLDMLLIATTEYSVGLPE-----ADGSSAGCKQTIPVKT----
[Mus                EGDVRPFIRFIAKCTEVTLDTLLLATTEYSVALPE-----AQPNHSGFKETLPVRP----
[Homo               EGDVRPFIRFIAKCTETTLDTLLFATTEYSVALPE-----AQPNHSGFKETLPVKP----
                     **:***:*::*. :* :::  : *       :            :   : :

[Caenorhabditis     --------------GN--
[Drosophila         AQRSSILEFYESGSGDLP
[Xenopus            ------------------
[Gallus             ------------------
[Mus                ------------------
[Homo               ------------------
```

Figure S1, relate to Figure 1. Sequence alignment of HYPE proteins from different species. Main structural elements, α-helices and flap, described in main Figures 1 and 2 are indicated in corresponding colors. Sequences corresponding to the inhibitory motif and catalytic loop are boxed.

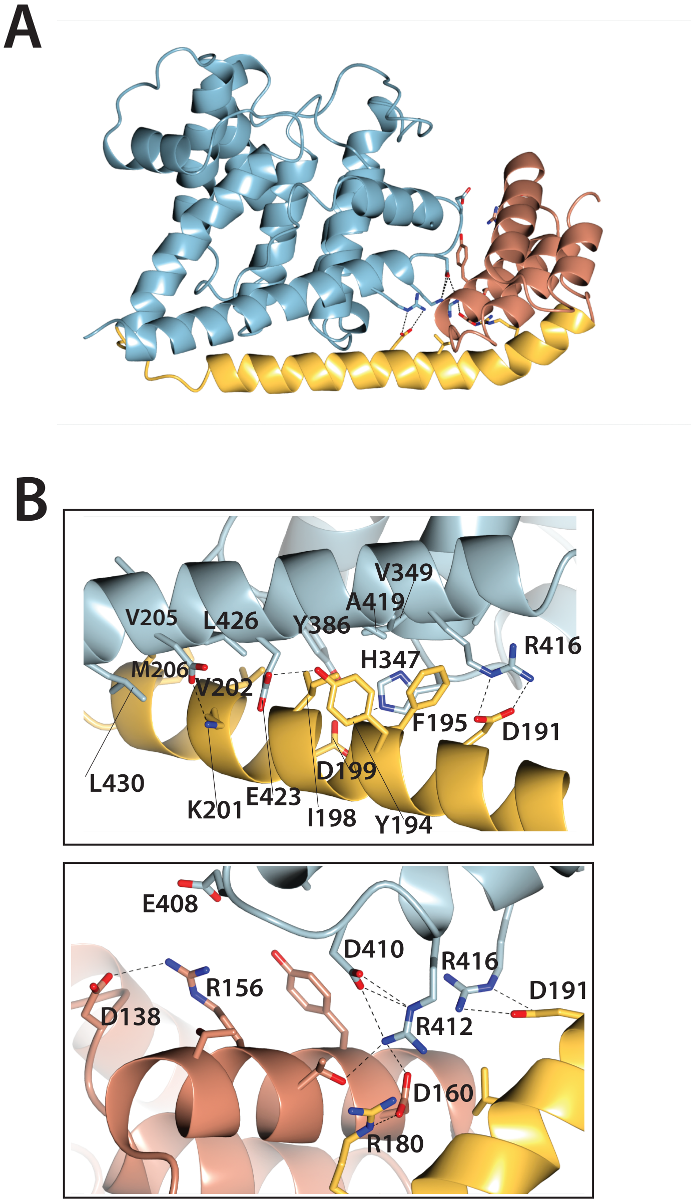

Figure S2, related to Figure 1. Surfaces of intra-molecular interactions
A. Ribbon representation of the HYPE construct; some of the residues involved in intra-molecular interactions are shown.
B. Residues involved in interactions between the FIC domain and linker are indicated in the top panel and residues contributing to interactions between the second TPR motif, FIC domain and linker are highlighted in the bottom panel.

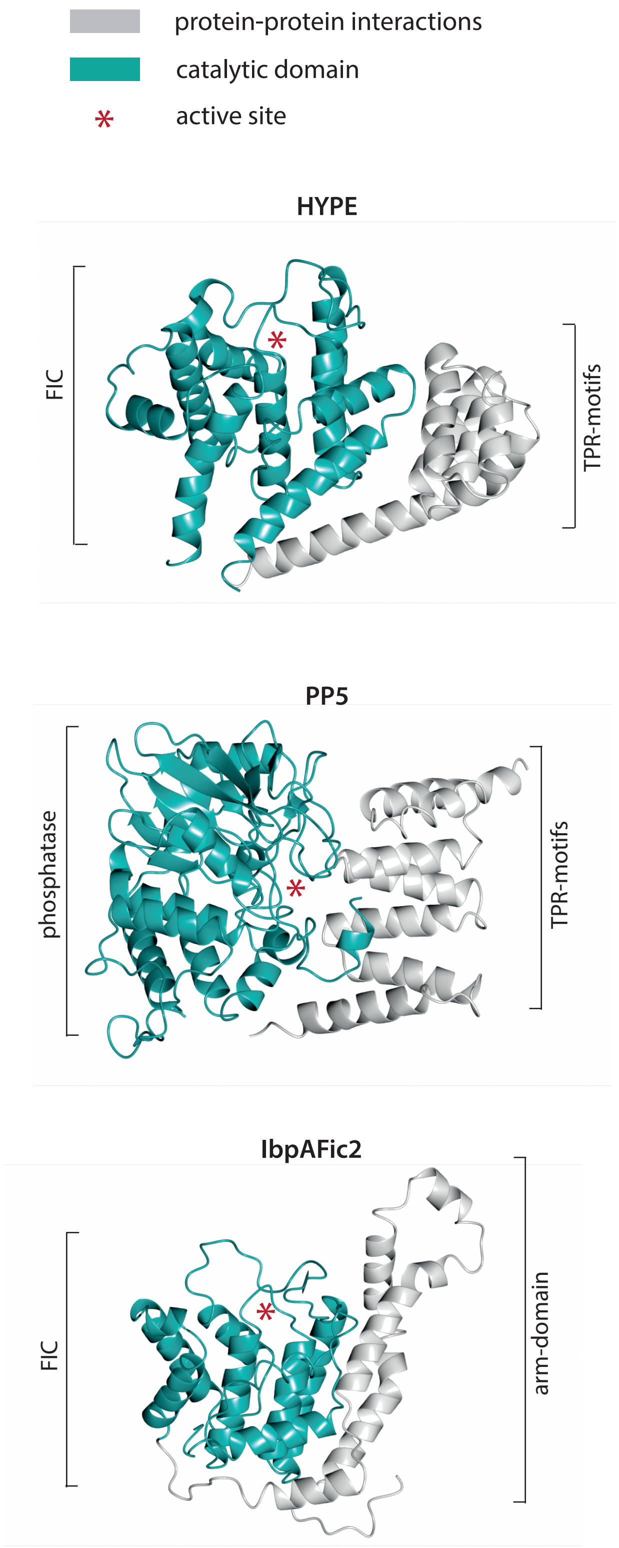

Figure S3, related to Figure 1. Inter-domain interactions in HYPE, PP5 and IbpAFic2
Relative orientation of the FIC domain and TPR-motifs in HYPE (top) is compared to orientation of phosphatase domain and TPR-motifs in PP5 (middle) and the FIC and arm domains of IbpAFic2 (bottom).
* indicates active site.

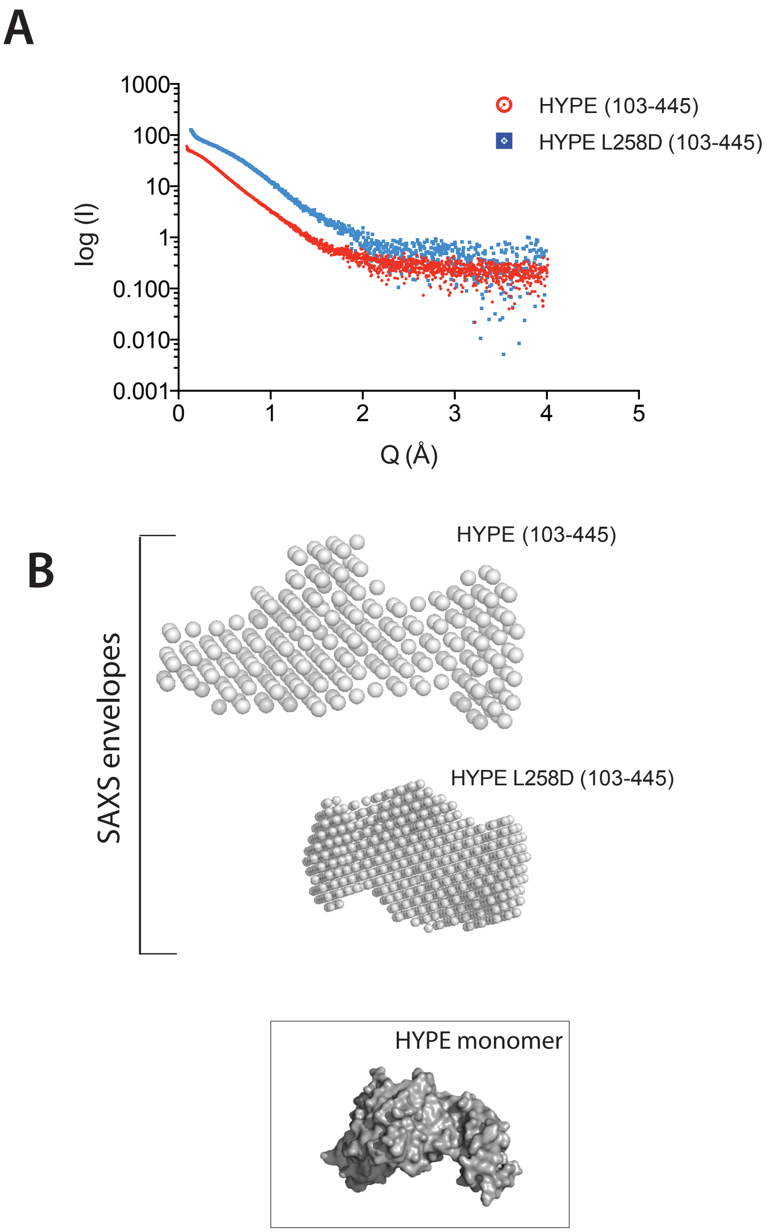

Figure S4, related to Figure 4. SAXS-based solution structure modeling of HYPE proteins
A) Log of scattered intensity versus Q for the experimental SAXS data of wild type HYPE (red circles) and the L258D variant (cyan squares).
B) DAMMIN generated envelopes of the wild type HYPE construct and L258D variant (top). Surface representation of the crystal structure of HYPE monomer is shown using the same relative scale as for the envelopes (bottom).

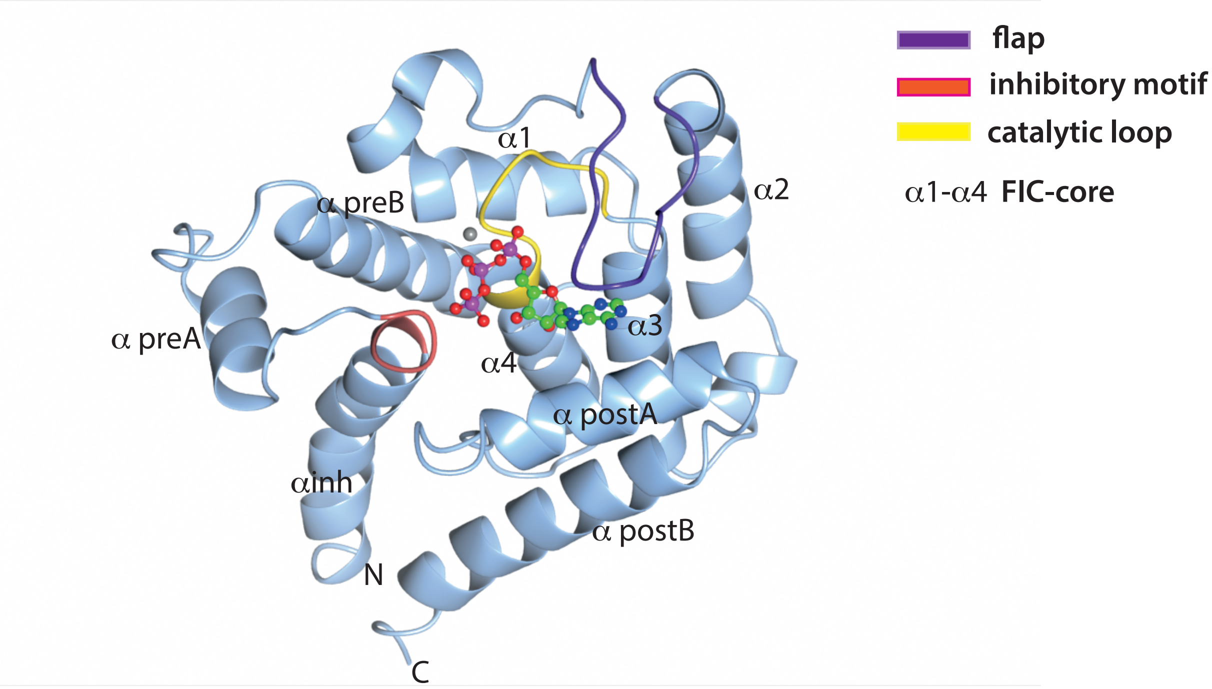

Figure S5, related to Figure 2. Features of FIC domain from HYPE
Structure of the FIC domain with highlighted catalytic loop, flap and inhibitory motif. ATP binding to E234G variant is shown; with respect to other structural features, no other changes outside the ATP binding site were observed in the corresponding structure, lacking this mutation.

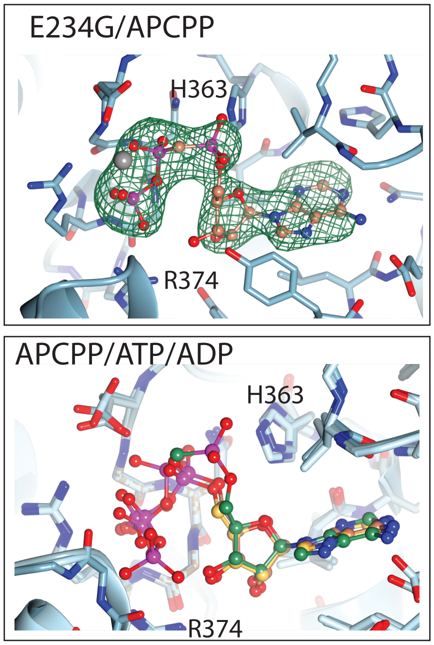

Figure S6, related to Figure 5. Binding site of APCPP
A) Binding pocket for APCPP in the E245G HYPE variant; density of APCPP, catalytic His 363 and γ-phosphate coordinating Arg 374 are indicated.
B) Overlay of ATP, ADP and APCPP in the binding pocket of E234G variant

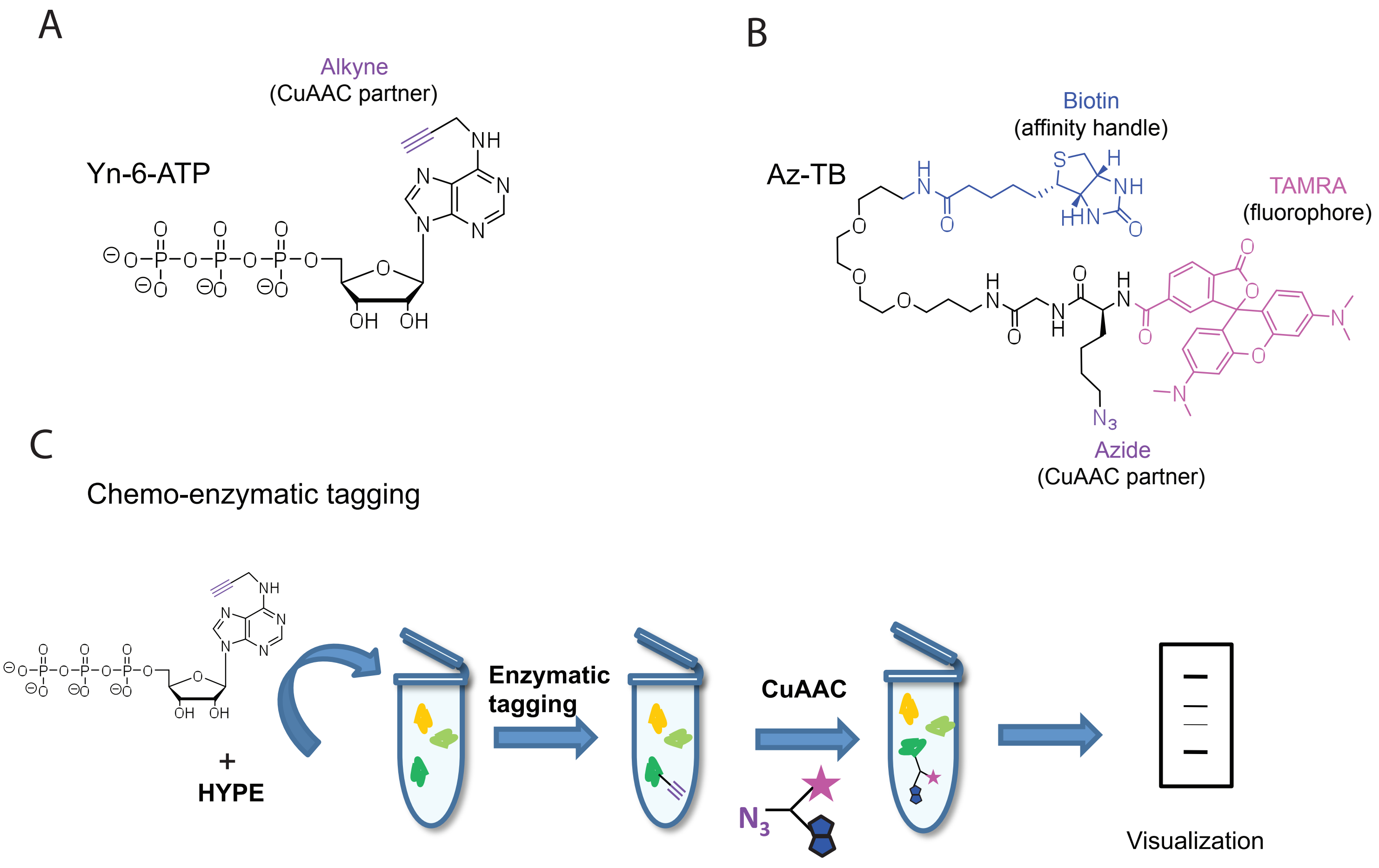

Figure S7, related to Figure 7. AMPylation assay
A) Structure of alkyne tagged adenosine triphosphate analogue Yn-6-ATP
B) Structure of multi-label reagent Az-TB used in CuAAC reaction; the fluorophore (TAMRA) permits direct in-gel detection, whilst biotin enables enrichment of AMPylated targets on streptavidin-agarose beads.
C) Auto-AMPylation or AMPylation of target proteins results in their tagging; the tagged proteins are ligated by copper-catalysed azide-alkyne cycloaddition (CuAAC) to a multi-label reporter that allows enrichment and visualization.

Supplemental Experimental Procedures

Western blotting of full-length HYPE constructs expressed in HEK 293F cells

Transfected Freestyle 293 cells were pelleted in 1 ml aliquots and the protein extracted in 0.25 ml of 25 mM Tris.Cl, 150 mM NaCl, 1 mM TCEP, 1 %(v/v) Triton-X-100, pH 7.5 containing a Complete protease inhibitor tablet (Roche). Protein concentration were determined following the BCA method and 30 µg loaded on an SDS-PAGE minigel. Proteins were transferred to PVDF membrane and blotted with an antibody against AMP-Threonine (Millipore). Blots were developed using ECL and film.

Small angle X-ray scattering (SAXS)

Synchrotron SAXS data were collected at ESRF (Grenoble). All samples were measured in 25 mM Tris.Cl, 150 mM NaCl, 5 mM TCEP, 10 %(v/v) glycerol at pH 8.0. Proteins were measured at 1, 2 and 5 mg/ml concentrations. The data were processed by the program PRIMUS (Konarev et al., 2003) following standard procedures to compute the radii of gyration (Rg) and maximum dimensions (Dmax) (Svergun and Koch, 2002). The distance distribution functions, p(r), were evaluated using the program GNOM (Svergun, 1992). The SAXS data for the monomeric mutant showed evidence of the formation of large aggregates apparent at lower q-values. We therefore excluded the points before the linear guiner region and used the rest of the curve for further modelling.

Low-resolution models were generated by the *ab initio* program DAMMIN (Svergun, 1999), which represents a protein by volume filled with the packed spheres referred to as dummy atoms. The results of 10 independent DAMMIN runs were analyzed and averaged by DAMAVER (Volkov and Svergun, 2003) and overlapped to the available crystal structure with the program SUPCOMB (Kozin and Svergun, 2001).

AMPylation assays

Cell culture and lysis. HeLa cells were cultured in a humidified atmosphere of 10% CO_2 at 37° C in DMEM supplemented with 10% FBS. Prior to lysis cells were washed twice with PBS and

harvested into the lysis buffer (20 mM Hepes pH 7.4, 100mM NaCl, 5 mM $MgCl_2$, 1% Triton X-100) supplemented with EDTA-free protein inhibitor (Roche). Next, the cells were disrupted by sonication and cellular debris was pelleted by centrifugation (17,000 g, 10 min). Protein concentration was measured with BCA assay (BioRad).

AMPylation reaction. Auto-AMPylation of recombinant HYPE variants (2 µg each) was carried out in the lysis buffer supplemented with BSA (2 µg), DTT (1 mM) and Yn-6-ATP (100 µM; Jena Bioscience) for 1 h at 30° C. AMPylation in cell lysates (50 µg protein) was performed using recombinant AMPylators (5 µg) as described above. AMPylation was stopped by protein precipitation (chloroform/methanol, 0.25:1, relative to the sample volume). Precipitates were isolated by centrifugation (17, 000 g, 10 min), washed once with methanol (400 µL) and air dried (10 min). Dried pellets were then resuspended (PBS, 0.2% SDS) at 1 mg/mL for the 'click' reaction.

CuAAC and in-gel fluorescence. A click mixture was prepared by adding reagents in the following order and by vortexing between the addition of each reagent: Az-TB (0.1 mM), $CuSO_4$ (1 mM), TCEP (1 mM), TBTA (0.1 mM). Az-TB was synthesized as described in Heal et al. (Heal et al., 2012). Following the addition of the click mix, samples were vortexed at RT for 1 h. The reaction was stopped by addition of EDTA (final concentration 10 mM) and subsequently proteins were precipitated, washed and dried as described above. The dried pellets were resuspended (PBS, 2 % SDS) and 4× LDS sample loading buffer (Invitrogen) was added (final concentration of proteins 1 mg/mL). The samples were then boiled (5 min), centrifuged (1,000 g, 2 min) and loaded on SDS-PAGE gel. After the run, the gel was washed with MilliQ (3x), soaked in fixing solution (40 % MeOH, 10 % acetic acid, 50 % water) for 5 min and washed with MilliQ (3x) again. The fluorescence on the gel was detected using an Ettan DIGE Imager (GE Healthcare) and the protein loading was checked by Coomassie.

Supplemental References

Heal, W.P., Wright, M.H., Thinon, E., and Tate, E.W. (2012). Multifunctional protein labeling via enzymatic N-terminal tagging and elaboration by click chemistry. Nat Protoc *7*, 105-117.

Konarev, P.V., Volkov, V.V., Sokolova, A.V., Koch, M.H.J., and Svergun, D.I. (2003). PRIMUS: a Windows PC-based system for small-angle scattering data analysis. J Appl Crystallogr *36*, 1277-1282.

Kozin, M.B., and Svergun, D.I. (2001). Automated matching of high- and lowresolution structural models. J Appl Crystallogr *34*, 33-41.

Svergun, D.I. (1992). Determination of the Regularization Parameter in Indirect-Transform Methods Using Perceptual Criteria. J Appl Crystallogr *25*, 495-503.

Svergun, D.I. (1999). Restoring low resolution structure of biological macromolecules from solution scattering using simulated annealing. Biophys J *76*, 2879-2886.

Svergun, D.I., and Koch, M.H. (2002). Advances in structure analysis using small-angle scattering in solution. Curr Opin Struct Biol *12*, 654-660.

Volkov, V.V., and Svergun, D.I. (2003). Uniqueness of ab initio shape determination in small-angle scattering. J Appl Crystallogr *36*, 860-864.
